# Direct interaction between marine cyanobacteria mediated by nanotubes

**DOI:** 10.1126/sciadv.adj1539

**Published:** 2024-05-23

**Authors:** Elisa Angulo-Cánovas, Ana Bartual, Rocío López-Igual, Ignacio Luque, Nikolai P. Radzinski, Irina Shilova, Maya Anjur-Dietrich, Gema García-Jurado, Bárbara Úbeda, José Antonio González-Reyes, Jesús Díez, Sallie W. Chisholm, José Manuel García-Fernández, María del Carmen Muñoz-Marín

**Affiliations:** ^1^ Departamento de Bioquímica y Biología Molecular, Campus de Excelencia Internacional Agroalimentario, Universidad de Córdoba, Córdoba 14014, Spain.; ^2^ Instituto Universitario de Investigaciones Marinas (INMAR), Campus de Excelencia Internacional del Mar (CEI·MAR), Universidad de Cádiz, 11510 Puerto Real, Cádiz, Spain.; ^3^ Instituto de Bioquímica Vegetal y Fotosíntesis, Consejo Superior de Investigaciones Científicas and Universidad de Sevilla, 41092 Sevilla, Spain.; ^4^ Department of Civil and Environmental Engineering, Massachusetts Institute of Technology, Cambridge, MA, USA.; ^5^ Independent researcher, St. Louis, MO, USA.; ^6^ Instituto Maimónides de Investigación Biomédica de Córdoba (IMIBIC), Córdoba, Spain.; ^7^ Departamento de Biología Celular, Fisiología e Inmunología, Campus de Excelencia Internacional Agroalimentario, Universidad de Córdoba, Córdoba 14014, Spain.

## Abstract

Microbial associations and interactions drive and regulate nutrient fluxes in the ocean. However, physical contact between cells of marine cyanobacteria has not been studied thus far. Here, we show a mechanism of direct interaction between the marine cyanobacteria *Prochlorococcus* and *Synechococcus*, the intercellular membrane nanotubes. We present evidence of inter- and intra-genus exchange of cytoplasmic material between neighboring and distant cells of cyanobacteria mediated by nanotubes. We visualized and measured these structures in xenic and axenic cultures and in natural samples. We show that nanotubes are produced between living cells, suggesting that this is a relevant system of exchange material in vivo. The discovery of nanotubes acting as exchange bridges in the most abundant photosynthetic organisms in the ocean may have important implications for their interactions with other organisms and their population dynamics.

## Teaser

Nanotubes mediate direct interactions in marine cyanobacteria.

## INTRODUCTION

The marine cyanobacteria *Synechococcus* and *Prochlorococcus* are responsible for approximately one-fourth of the primary production in the ocean (*1*). Their high abundance in oligotrophic areas of the oceans is the result of the diverse strategies they have developed to acquire and economically use nutrients. Other marine bacteria have evolved various mechanisms of interaction with the ocean and neighboring cells (*2*–*6*) to enhance nutrient acquisition. Bacterial nanotubes are membrane-coated tubular structures connecting two cells that allow the transport of cytoplasmic components between them. In bacteria, nanotubes were first identified in *Bacillus subtilis* (*7*, *8*) and *Escherichia coli* (*7*) and, recently, in marine heterotrophic bacteria (*9*). Intercellular nanotubes can function as conduits for transporting metabolites (e.g., amino acids), proteins (including toxins), and nonconjugative plasmids (*7*, *10*–*12*). They can also play critical structural functions in biofilm formation on patterned surfaces (*7*). Similar to nanowires (*13*) and chains of phospholipid vesicles (*14*), nanotubes might facilitate nutrient uptake in small microorganisms living in oligotrophic conditions, such as in the epipelagic open ocean, by increasing their surface-to-volume ratio.

We observed the occurrence of nanotubes in pictures of marine cyanobacteria obtained with several microscopy techniques. We then visualized and measured these structures by combining different techniques in xenic and axenic laboratory cultures and in natural samples. We also investigated the inter- and intra-genus exchange of cytoplasmic material between neighboring and distant cells of the cyanobacteria *Prochlorococcus* and *Synechococcus* mediated by nanotubes. Last, we studied the possible key genes involved in nanotube production. The finding that marine cyanobacteria can exchange material with each other by direct interaction using nanotubes has important implications for the evolution and ecology of microbial life in the open ocean.

## RESULTS AND DISCUSSION

### Nanotubes are identified in marine cyanobacteria

**Fig. 1.**
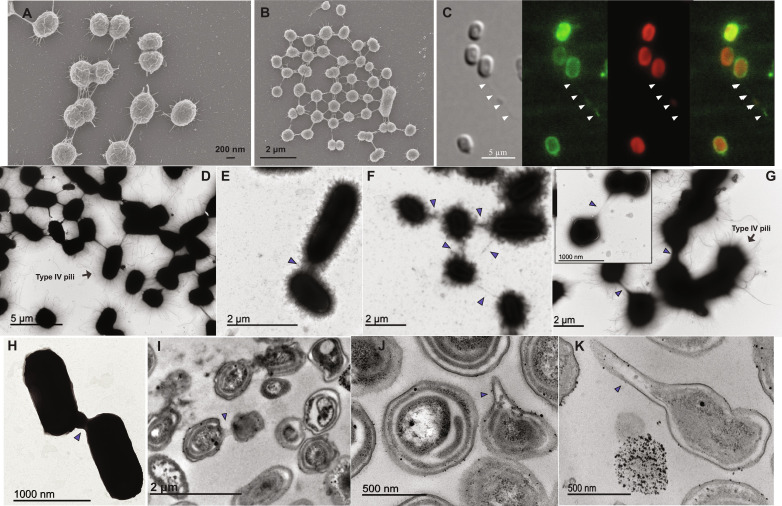
Nanotubes form between cyanobacterial cells. (**A** and **B**) SEM images of *Prochlorococcus* sp. SB (xenic culture grown in coculture with *Alteromonas*). (**C**) Fluorescence images of *Synechococcus* sp. PCC 7002 stained with the lipophilic fluorescent dye FM 1-43. Three cells and one nanotube imaged by bright field (left), membranes stained by FM 1-43 (green), cyanobacterial autofluorescence (red), and merged images (right). (**D** to **H**) Transmission electron micrographs of axenic cultures of *Synechococcus* sp. PCC 7002 (D), sp. WH8102 (E), and sp. WH7803 (F and H) and *Prochlorococcus* sp. MIT9313 (G). (**I** to **K**) Ultrathin sections of *Synechococcus* sp. WH7803 by TEM. Arrows indicate intercellular nanotubes connecting neighboring cells. (A) to (D), (F), and (G) show narrow nanotubes, and (E), (G), and (H) to (K) show wide nanotubes. Type IV pili are shown in micrographs (A), (B), and (D) to (G).

While examining a sample of *Synechococcus* sp. PCC 7002 using transmission electron microscopy (TEM), we noticed the presence of appendages, which we hypothesized were nanotubes connecting several cells. We also observed similar structures in cultures of marine *Synechococcus* [strains WH8102 (Marine A III) and WH7803 (Marine A V) (*15*)] and *Prochlorococcus* [strains SB (high light–adapted ecotype II), MIT9313 (low light–adapted ecotype IV), and SS120 (low light–adapted ecotype II) (*16*)], which are not known to form conjugative pili (*17*). A combination of TEM, scanning electron microscopy (SEM), fluorescence microscopy, and imaging flow cytometry (IFC; using the Amnis ImageStream MKII system, Luminex) was used to observe and characterize nanotubes in several xenic and axenic cultures of *Prochlorococcus* and *Synechococcus* (Fig. 1 and fig. S1). The electron microscopy revealed that nanotubes project from the cell surface at different positions, each mediating a connection with another cell, so that one cell can be connected to several others simultaneously (Fig. 1, A, B, D, F, and G). Growth conditions (e.g., solid or liquid media and agitated or not agitated) had no effect on the formation of nanotubes. We also exclude the possibility that nanotubes are by-products of the cell fixation procedure because the fluorescence microscopy and the IFC approaches required no cell fixation (unlike SEM and TEM). We also discarded the possibility that cell-cell connections were artificially produced from samples concentrated by centrifugation (as required by TEM) or grown on soft agar plates. To this end, we analyzed planktonic cells and also grew cells to naturally promote adherence as recently found by Capovilla *et al.* (*18*). Our results showed that any mechanism that brings cells much closer than they would be in nature does not affect nanotube formation (see Supplementary Text and fig. S2).

Nanotubes were observed in cultures grown to promote natural adherence using SEM and TEM (Fig. 1, A and B, and fig. S3). Observation of these cell-cell connections in planktonic cells by the same technique was more difficult given how dilute picocyanobacterial cultures are. Nevertheless, we were able to observe a few cells connected by nanotubes in different planktonic xenic and axenic strains of *Prochlorococcus* and *Synechococcus* (fig. S4). Furthermore, the use of IFC of free-floating cells also provided convincing evidence that nanotubes are produced in planktonic cells since this also does not require concentration of the cells before observation (see Materials and Methods).

Two types of cyanobacterial nanotubes were observed by TEM varying in width: wide (100 to 200 nm) and narrow (<100 nm) (Supplementary Materials). A cylindrical structure with such internal diameter is calculated to allow the passage of small molecules as calcein [623 Da (*19*)] in narrow nanotubes, green fluorescent protein (GFP) [26.8 kDa (*20*)], and even larger molecules in wide nanotubes. The nanotube length reached up to 0.5 μm (e.g., Fig. 1, E and H), although we occasionally observed longer appendages connecting two cells (up to 1 μm) (e.g., Fig. 1, B and D). Overall, the size of nanotubes in marine cyanobacteria was consistent with the sizes previously described in bacteria (*7*). These appendages were distinct from those described for the type IV pili (*21*), which measured approximately 6 nm in diameter and 10 μm in length (Fig. 1, D to G) as reported by Aguilo-Ferretjans *et al.* (*21*).

Connections by these structures were observed between neighboring cells (located ≤2 μm from other cells) but also between distant cells (located >2 μm away from other cells) (e.g., Fig. 1, A, B, D, F, and G). Rarely, we observed individual cells with the nanotubular structures unconnected to other cells, which could suggest their recent separation, or that the nanotube formation occurred before the approach to a neighboring cell (Fig. 1, J and K).

To check whether these intercellular nanotubes consist of membrane-derived lipids as described previously for bacterial nanotubes (*9*, *11*), we labeled *Synechococcus* cells with the lipophilic fluorescent dye FM 1-43 {*N*-(3-triethylammoniumpropyl)-4-[4-(dibutylamino) styryl] pyridinium dibromide} used to stain cell membranes in eukaryotic and prokaryotic cells (*22*). Only the cell membrane and the nanotube were labeled by the lipophilic stain, suggesting that the nanotubes have a lipidic composition (Fig. 1C) and are distinct from conjugative pili, which are mainly composed of proteins (*23*). Moreover, ultrathin section analysis using TEM suggests that nanotubes contain membrane and cytoplasmic components (Fig. 1, I to K). Thus, based on these characteristics, the identified appendages appear to be true bacterial nanotubes.

**Fig. 2.**
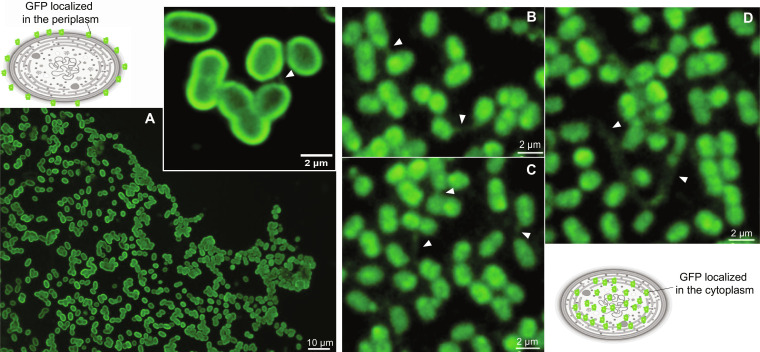
Nanotubes connecting *Synechococcus* sp. PCC 7002 recombinant cells expressing sfGFP. (**A**) A field of recombinant cells with pGFP. Top: An additional example of magnified cells showing the pGFP recombinant cells. (**B** to **D**) Recombinant cells with the cGFP. White arrows indicate the localization of GFP molecules within the nanotubes. The *Synechococcus* cells drawn in the upper left and lower right corners show the two different sfGFP locations used in the experiment.

**Fig. 3.**
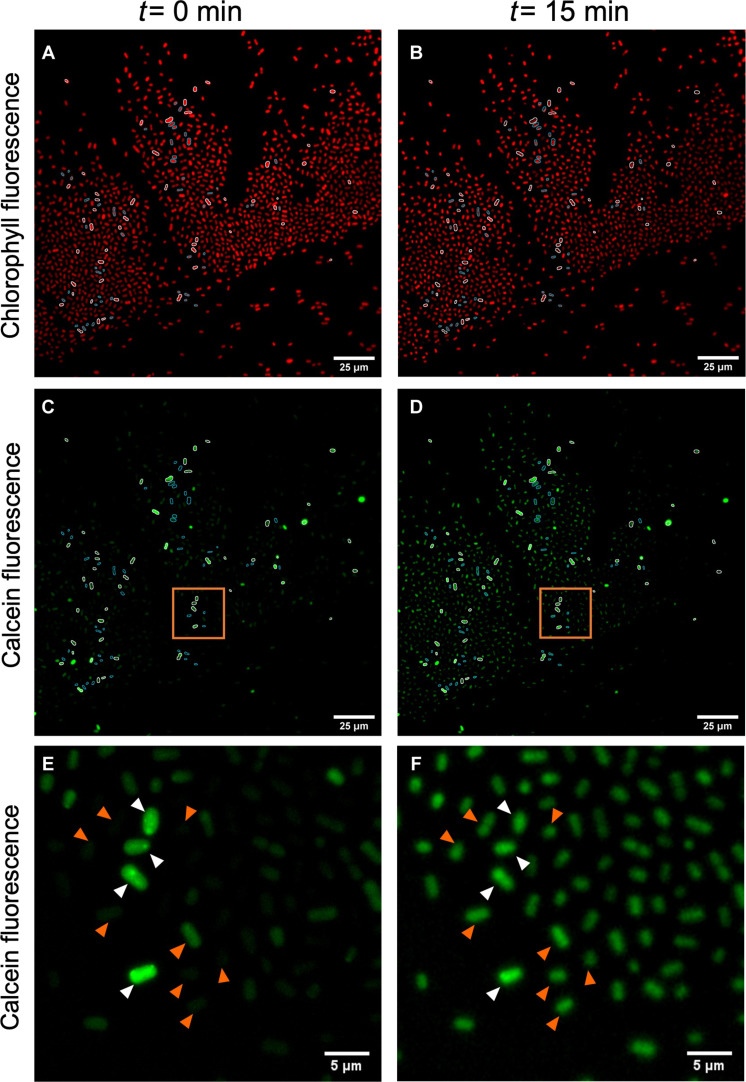
Transfer of calcein between *Synechococcus* sp. PCC 7002 cells. (**A** to **D**) *Synechococcus* sp. PCC 7002 calcein-labeled cells were mixed with unlabeled ones. A total of 100 cells were selected: 50 calcein-labeled cells (white circle) and 50 unlabeled cells (cyan circle). The average fluorescence intensity at *t* = 0 min versus *t* = 15 min (after the mix) was compared (Supplementary Materials and table S1). (**E** and **F**) Magnification of the orange squares in (C) and (D). White arrows highlight cells whose average fluorescence intensity decreases after 15 min. Orange arrows highlight cells whose average fluorescence intensity increases after 15 min.

**Fig. 4.**
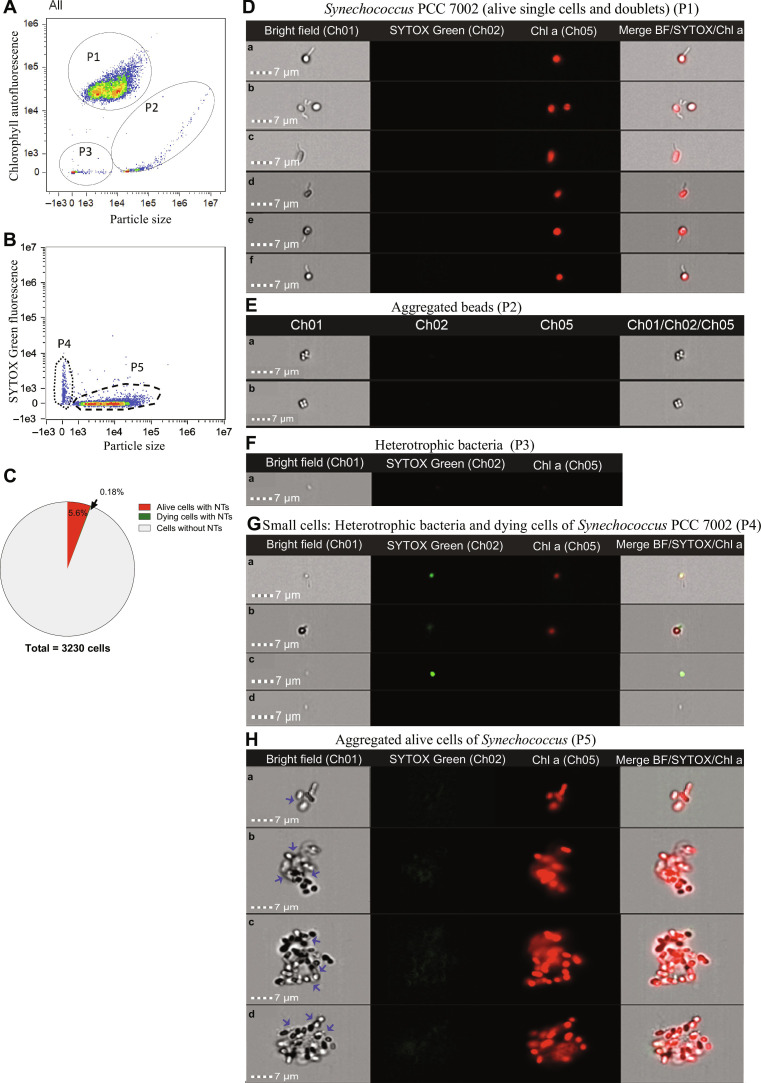
Correlation between nanotube formation and vitality in xenic cultures of *Synechococcus* sp. PCC 7002 observed by IFC. (**A**) Cytogram showing *Synechococcus* populations observed in cultures. The particle size and chlorophyll autofluorescence identified alive single or doublets *Synechococcus* cells (subpopulation P1), aggregated beads (subpopulation P2), and cellular debris with heterotrophic bacteria (subpopulation P3). (**B**) Cytogram obtained from SYTOX Green–stained samples. The SYTOX Green fluorescence allowed to distinguish between alive or dead cells. Subpopulation P4 includes small particles like heterotrophic bacteria (alive or dead) and dying cells of *Synechococcus*, and subpopulation P5 includes large size particles, which are alive and dead *Synechococcus* cells (solitary or into aggregates). (**C**) Pie chart showing the percentage of alive cells and damaged cells with nanotubes (NTs) from a culture population of 3230 cells (see Materials and Methods). (**D**) Bright-field images at ×60 magnification from *Synechococcus* alive cells [chlorophyll a (Chl a) autofluorescence; a to f]. (**E**) Bright-field images at ×60 magnification from aggregated calibration beads (larger size and non–Chl a autofluorescence; a and b). (**F**) Bright-field images at ×60 magnification from heterotrophic bacteria alive cells (smaller size and non–Chl a autofluorescence; a). (**G**) Bright-field images at ×60 magnification from small particles including *Synechococcus* dying cells (Chl a autofluorescence and SYTOX Green fluorescence; a and b), dying heterotrophic bacteria (SYTOX Green fluorescence and non–Chl a autofluorescence; c), and alive heterotrophic bacteria (non–Chl a autofluorescence; d). (**H**) Bright-field images of aggregated alive cells of *Synechococcus* (Chl a autofluorescence; a to d). Blue arrows show nanotubes in aggregated cells (see also fig. S18).

Why have nanotubes never been previously observed in marine cyanobacteria? Images of putative nanotubes (or nanotube-like structures) have appeared in early publications on *Synechococcus* [figures 1, 2, and 4 of (*24*) and figure 3 of (*25*)] and *Prochlorococcus* [figure 1A of (*2*)], but the authors did not mention them in these papers. The uncertainty of whether they were true structures, artifacts of the cell fixation procedure, or extracellular cellulose in the case of some *Synechococcus* (*26*) may have contributed to the lack of attention to these appendages for decades. Moreover, the nanotubes are not easily distinguished by bright-field or phase-contrast microscopy, and they rarely display autofluorescence, which makes their identification more difficult. Differences in sample preparation can also affect the reliability of nanotube observations under the microscope. The TEM laser or the fixation methods seem to break the thinner connections in our samples. Such a discovery of a previously overlooked structure has happened before in marine cyanobacterial cultures. Biller *et al.* (*2*) found the presence of marine vesicles in the ocean. These authors reported that these small packages of information were secreted by the cells to the ocean, playing a possible role in communication between cells (*2*). Now, it is rare not to observe *Prochlorococcus* and *Synechococcus* vesicles in any previous electron microscopy images. We hypothesize that something similar is occurring regarding cyanobacterial nanotubes, which, although appearing at very low frequencies in our samples, are easily identified as being a regular feature of these systems.

### Nanotubes transfer cytoplasmic molecules

We explored if nanotubes in cyanobacteria were produced by the protrusion/blebbing of the outer membrane, enabling the exchange only of periplasmic content (*22*), or if they were coated by plasma and the outer membrane as concentrical cylinders enclosing cytoplasmic and periplasmic spaces (*8*). For this, *Synechococcus* PCC 7002 was transformed with either of two plasmids: one encoding a periplasmic version of the GFP (pGFP) and another one encoding cytoplasmic GFP (cGFP). As expected, the cyanobacterial strains carrying pGFP showed a high signal in the periphery of the cells, although nanotubes were hardly observed (Fig. 2A). In contrast, in the strains with cGFP, nanotubes were readily identified with sizes that ranged from ~0.3 to 2.3 μm (Fig. 2, B and C), in agreement with the hypothesis that the nanotubes connect cell cytoplasms rather than just the periplasmic spaces. The weak pGFP signal in the nanotubes was due to either their minute periplasmic volume, considerably smaller than the inner cytoplasmic space, or restricted diffusion of periplasmic content in the nanotube.

Rarely, we found nanotubes longer than ~18 μm connecting distant cells (Fig. 2D). The width barely changed, measuring ~0.6 ± 0.1 μm (*n* = 11), slightly exceeding the size observed in the SEM and TEM images. It should be noted that the maximum resolution of fluorescence microscopy made it difficult to accurately measure the width of nanotubes. Moreover, visualization of narrow nanotubes by fluorescence microscopy was challenging. Our results indicate a cytoplasmic connection between cells, suggesting that cGFP can move along the connection, demonstrating the capacity of nanotubes to transfer molecules between cells.

To confirm that nanotubes facilitate transfer of cytoplasmic molecules from a donor cell to recipient cells in cyanobacteria, we used calcein, an artificial fluorescent tracer (fig. S5) (*19*). The nonfluorescent calcein acetoxymethyl ester (AM) permeates into cells, where, once converted by cytoplasmic esterases into fluorescent calcein (excitation/emission, 494/517 nm), it remains trapped due to its nonpermeable nature (*19*, *27*). When *Synechococcus* sp. PCC 7002 cells were incubated with calcein-AM, the cells acquired a strong fluorescence signal within 3 hours of incubation (fig. S6, A and B), in contrast to cells not incubated with calcein (fig. S6, C and D). The labeled cells were washed five times to remove any remaining calcein-AM from outside the cells and mixed with nonlabeled cells (1:1 ratio), and the mixture was tracked by time-lapse fluorescence microscopy for 15 min (Fig. 3). Over this period, the nonlabeled cells displayed an increase in the fluorescence signal, while fluorescence decreased in previously labeled cells (Fig. 3, C and D, and table S1). After 15 min, almost all the nonlabeled cells (>80%) showed fluorescence, suggesting the transfer of calcein between neighboring cells (Fig. 3). In contrast, a parallel control of unlabeled cells incubated for 15 min in the presence of the supernatant of washed labeled cells (processed as in the experiment above) showed no fluorescence (fig. S6, E and F), confirming that the medium of washed labeled cells was free of calcein-AM. Thus, the fluorescence increase in unlabeled cells in the mixing experiment was due to the transfer of calcein from donor cells. One last control was included in which the labeled donor cells were killed before allowing them to interact with recipient cells, showing that active exchange is needed in this process (fig. S7).

We also investigated whether nanotubes could form between different genera of marine cyanobacteria. Cultures of two marine cyanobacteria easily distinguishable under the fluorescence microscope were used: the rod-shaped relatively large cells (~1 to 3 μm) of *Synechococcus* sp. PCC 7002 and the cocci-shaped small cells of *Prochlorococcus* sp. MIT9313 and SS120 (~0.5 to 0.7 μm). Notice that, in these experiments, the average calcein fluorescence intensity per cell varied between *Synechococcus* and *Prochlorococcus*, likely due to different cell volumes of *Prochlorococcus* (*28*) and *Synechococcus* (*29*). *Synechococcus* sp. strains WH7803 and WH8102 were not used in this experiment as they contain phycoerythrin and phycocyanin, which interfere with the calcein signal (fig. S8). *Synechococcus* sp. PCC 7002 cells labeled with calcein-AM were mixed (1:1 ratio) with unlabeled *Prochlorococcus* sp. MIT9313. After 15 min of incubation, *Prochlorococcus* cells showed increased green fluorescence, while *Synechococcus* cells showed a decrease in calcein fluorescence (fig. S9, C and D, and table S1). A few cells of *Prochlorococcus* showed a decrease in green fluorescence before 15 min, likely because the calcein exchange occurred in a short time period (fig. S9, C and D). The loss of signal also occurred when *Synechococcus* sp. PCC 7002 labeled cells were mixed with *Synechococcus* sp. PCC 7002 nonlabeled cells (Fig. 3).

**Fig. 5.**
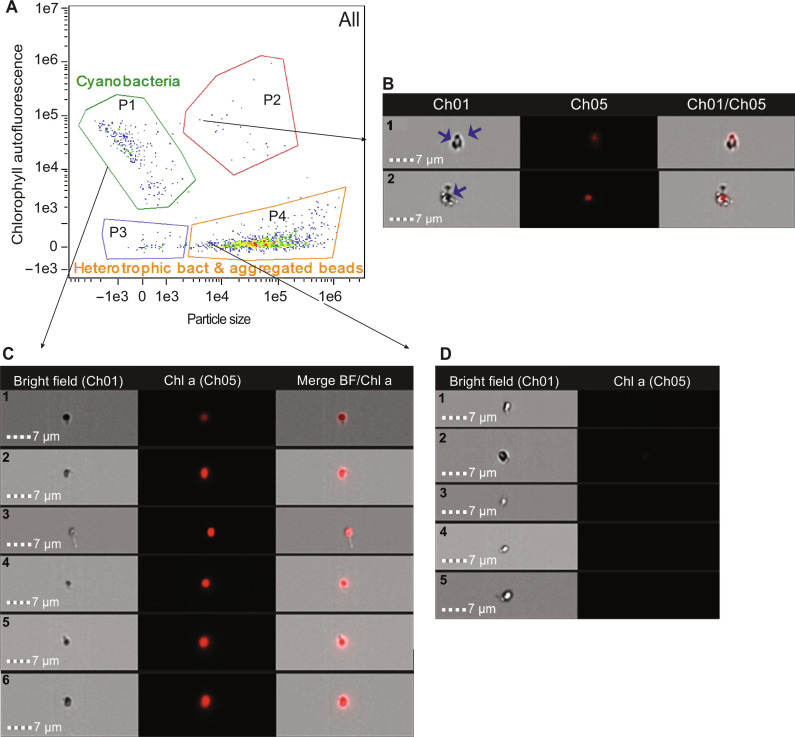
Nanotube formation in natural population of cyanobacteria observed by IFC. (**A**) Cytogram obtained from the sample collected on 7 March 2023 by IFC showing the cyanobacterial population discerned by size and pigment composition (Chl a) (P1), aggregates of cyanobacterial cells (Chl a autofluorescence in the small particle area) and photosynthetic eukaryotes (Chl a autofluorescence in the large particle area) (P2), detritus and some heterotrophic bacteria (P3), and heterotrophic bacteria and aggregated beads (P4). P3 and P4 populations might also include some cyanobacterial and picoeukaryotic dead cells. (**B**) Bright-field images at ×60 magnification of aggregates of *Synechococcus* cells (Chl a autofluorescence) with other cells (dying cyanobacterial cells or heterotrophic bacteria; a and b). Blue arrows show nanotubes in aggregated cells. (**C**) Bright-field images at ×60 magnification of individual *Synechococcus* cells (Chl a autofluorescence; a to f). (**D**) Bright-field images at ×60 magnification of individual heterotrophic bacteria (a to e).

Similar results were observed (fig. S10) when calcein-labeled *Prochlorococcus* sp. SS120 cells (fig. S11B) were mixed with unlabeled recipient *Synechococcus* sp. PCC 7002 cells (fig. S6D). We studied the fluorescence intensity of cells in the mixed population by time-lapse microscopy, determining in which time frame each cell showed its maximum fluorescence. As expected, *Prochlorococcus* cells (calcein-labeled) displayed their maximal fluorescence at early time points (<2 min). In contrast, most of the nonlabeled *Synechococcus* sp. PCC 7002 cells reached their maximum fluorescence after 6 min of incubation (see Supplementary Text and figs. S12 and S13). Overall, these results indicate inter-genera molecular transfer within a relatively short time, less than 15 min (table S1). This is consistent with previous studies showing that bacterial nanotubes connect two cells of the same (*12*) or different bacterial species (*30*) and with eukaryotic cells (*31*). We also observed a few interactions mediated by nanotubes between cyanobacteria (*Prochlorococcus* or *Synechococcus*) and heterotrophic bacteria (Figs. 1B, 4, and 5 and fig. S14). Future efforts will elucidate if nanotubes occur in other marine cyanobacteria and heterotrophic bacteria.

Both cGFP and calcein experiments show that *Synechococcus* sp. PCC 7002 cells are able to exchange cytoplasmic molecules via nanotubes. The fact that nanotubes were not visualized in most of the calcein transfer experiments contrasts with the strong fluorescence of nanotubes in cells expressing cGFP (compare Figs. 2 and 3; see also fig. S12). This might be related to the size difference of these two tracers and their disparate diffusion rates. Since the diffusion rate of a molecule is inversely proportional to its molecular weight (*32*), the expected residence time of GFP (28.6 kDa) in the nanotubes would be much longer than that of calcein molecules (623 Da). Thus, while cGFP molecules would accumulate in nanotubes producing high fluorescence, the short residence time of calcein would preclude the accumulation of sufficient molecules to be detected.

### Nanotubes are formed from live cells

Moreover, we excluded the possibility that nanotubes were produced by dying cells as was observed in *B. subtilis* (*33*). Calcein-AM is used to determine cell viability in eukaryotic and prokaryotic cells (*34*–*38*). The fact that calcein-AM was hydrolyzed in cyanobacterial cells indicated that the cells were metabolically active. In addition, flow cytometry monitoring showed that cells remain alive for at least 3 hours after calcein addition (fig. S15). Furthermore, a negative control (cells killed by boiling) showed that dead cells did not hydrolyze calcein-AM after 3 hours of incubation (figs. S16 and S17).

To further confirm that nanotube-mediated transfer of cytoplasmic material occurs in active cells, we analyzed the presence of nanotubes in a culture of *Synechococcus* sp. PCC 7002 using SYTOX Green stain and the ImageStream MKII flow cytometer. SYTOX Green penetrates cells with compromised/permeabilized membranes where it stains nucleic acids, indicating cell death (*39*). By using the ImageStream MKII flow cytometer, we can identify the different populations present in the culture, distinguishing between live and dead or damaged cells (Fig. 4, A and B), and also analyze the presence of nanotubes in these cells (Fig. 4, D to H). Another advantage of using this instrument is that we eliminated the possibility that nanotubes were created due to the pressure applied by the coverslips (*33*) or the possibility that cell-cell connections were artificially produced from samples concentrated by centrifugation since such manipulations are not required in the preparation of samples for flow cytometry. We observed individual cyanobacterial cells with several nanotubes (Fig. 4D) and also aggregated live cells of *Synechococcus* connected by these nanotubes (Fig. 4H and fig. S18A). In laboratory cultures, most nanotubes were observed in living cyanobacteria (96.8% of the total nanotubes identified, corresponding to 5.6% of the total cells; see Fig. 4, C, D, and H). SYTOX Green was incorporated into the compromised cells, where the chlorophyll signal was lower. As in previous reported results (*33*), we seldomly observed damaged/dying cells showing nanotubes (3.2% of the total nanotubes identified, corresponding to 0.18% of the total cells Fig. 4, C and G). We also observed a few aggregates of dying and live cells with nanotubes between them (fig. S18B).

In summary, the results from calcein-AM and SYTOX Green experiments showed that nanotubes are mostly formed in metabolically active cells, at least for the cyanobacterial strains tested here. Cell viability as a requirement for nanotube formation was also reported in previous studies (*7*, *9*). However, the presence of a few nanotubes in aggregates of both dying and living cyanobacteria could suggest the transfer of nutrients from dying to live cells as a possible solution for their nutritional limitation in the oligotrophic ocean, favoring the aggregation of cells.

### Cyanobacterial nanotubes are present in natural population

Last, to explore the occurrence of cyanobacterial nanotubes in natural populations of cyanobacteria, we examined nonfixed samples from the estuarine system of Cádiz Bay (Spain) by using an imaging flow cytometer (Fig. 5). Figure 5 (B and C) reveals the presence of nanotubes in marine cyanobacteria (solitary or in aggregates). The cyanobacterial population was identified in the cytograms by size and pigment composition. The natural cyanobacteria cell size was much smaller than that of *Synechococcus* sp. PCC 7002, which made nanotube identification extremely difficult (Fig. 5, B and C).

In natural samples, a total of 282 cells of cyanobacteria were analyzed in triplicate on 7 March 2023 in different locations. An average of 5.5 ± 1.06% of cyanobacterial cells showed nanotubes (see Materials and Methods), a very similar percentage to that observed in cells of *Synechococcus* sp. PCC 7002 in culture (5.8% of the total cells showed nanotubes). These results show the presence of nanotubes in natural cyanobacterial populations, thus confirming that this is not a laboratory artifact but an appendage occurring in natural samples. We also observed, in these samples, the presence of nanotubes in the heterotrophic population, which has been recently described (Fig. 5D) (*9*).

### Cell wall hydrolases might be involved in nanotube formation

The genetic basis of nanotube formation has been described for two model bacteria (*8*, *10*) and includes the genes for the export apparatus [known as the CORE genes (*31*, *33*, *40*); table S2]. No hits were found in picocyanobacterial genomes for the CORE genes when doing a search by BLASTP (tables S3 to S5). Therefore, picocyanobacteria are likely to use an alternative system to build nanotubes. Putative homologs of the two major cell wall hydrolases (*lytB* and *lytC*) and peptidoglycan endopeptidase (*lytE*), associated with nanotube formation (*41*), were found in picocyanobacterial genomes (see Materials and Methods); however, it is unknown whether they are involved in nanotube formation and what other genes might play the function of CORE genes in picocyanobacteria.

Cyanobacterial nanotubes may play a critical ecological role in bacterial adaptation to the oligotrophic oceans. Whether nanotubes mediate unidirectional or bidirectional exchange will need to be investigated. However, our calcein transfer experiments between *Prochlorococcus* and *Synechococcus* showed that cells of both genera can act as either donors or recipients. The intercellular transfer of material observed in this work stirs a longstanding debate (*7*, *33*) on the benefit of this phenomenon for donor and recipient cells.

There are multiple studies suggesting that marine cyanobacteria and heterotrophic bacteria have co-dependent metabolisms rather than functioning solely as individual organisms (*2*, *42*–*44*), causing the establishment of cross-feeding interactions according to the “Black Queen Hypothesis” (*45*). The finding that marine cyanobacteria can exchange material with each other by direct interaction using nanotubes has important implications for the evolution and ecology of microbial life in the open ocean.

*Prochlorococcus* and *Synechococcus* are small; both have streamlined genomes, and most are nonmotile. Many of them thrive in poor nutrient areas, to which they have adapted by using different strategies as high-affinity uptake systems (*46*, *47*), the use of type IV pili for mobility in search for better nutrient conditions (*21*), the use of flagella by some *Synechococcus* to swim toward nutrient hotspots in the oceans (*48*), and, most commonly, by mutualism strategies (*49*, *50*). Increasing the surface-to-volume ratio by the formation of nanotubes, especially in the case of small cell sizes (i.e., one nanotube can increase the surface-to-volume ratio of a 2-μm cell by 39%; see Supplementary Text and fig. S19), or even connecting aggregated cells would be a solution for their nutritional limitation in the oligotrophic ocean, improving nutrient uptake opportunities for small microorganisms. Transport of metabolites, proteins, and genetic information has been seen in other bacterial nanotube studies (*7*, *10*–*12*) and is an intriguing target for potential investigation.

The relevance of these connections in the extremely dilute milieu of the oligotrophic oceans could be important under some specific conditions, for instance, in marine snow where the bacteria-bacteria separation on average is approximately 10 μm (*9*), in the small colloids (around 10^9^ particles per milliliter) (*51*), and in marine biofilms (*52*). Therefore, the actual impact of the nanotube-mediated exchange on natural populations would depend on the frequency of cell-cell interaction, which, in turn, depends on the concentration of cells, a largely variable parameter in distinct areas of the ocean. The observations of nanotubes in marine bacteria, including natural picocyanobacterial populations reported here and heterotrophic bacteria reported earlier (*9*), suggest that nanotubes might be a common interaction mechanism among microbes. However, further studies would be required to determine the global dimension of the transfer phenomenon as well as its relevance in particular areas.

## MATERIALS AND METHODS

### Growth of bacterial strains

Axenic *Prochlorococcus* strains MIT9313 and SB and xenic SS120 were routinely cultured in sterile polycarbonate flasks (50 or 250 ml; Nalgene) using PCR-S11 medium (*53*). We obtained the seawater used as basis for the PCR-S11 medium from the Mediterranean Sea, provided by the Instituto Español de Oceanografía (Spain). Cultures were grown in a culture room at 24°C under continuous blue irradiance [4 to 40 microeinstein (μE)/m^2^ s depending on the strain] using neon Sylvania F18W/154-ST Daylight, covered with a Moonlight blue L183 filter from Lee Filters. We used a BioMate 3S UV-Visible spectrophotometer (Thermo Fisher Scientific) to measure the absorbance of cultures at 674 nm to determine their growth, performing the experiments during the exponential phase of growth (*A*_674_ = 0.05).

Xenic and axenic *Synechococcus* strains sp. WH8102 and WH7803 were cultured in a culture room as described above for *Prochlorococcus* using RSS medium (*54*). We used a BioMate 3S UV-Visible spectrophotometer (Thermo Fisher Scientific) to measure the absorbance of cultures at 495 nm for strain WH8102 and 550 nm for strain WH7803 to determine their growth. We performed the experiments during the exponential phase of growth (*A*_495/550_ = 0.1).

Xenic and axenic *Synechococcus* sp. PCC 7002 was cultured in sterilized flasks in an Algaetron AG 230 growth chamber (Photon Systems Instruments) at 30°C, with white LED (light-emitting diode) light at 10 μE/m^2^ s and orbital shaking at 180 rpm, using a mixture (1:1 v/v) of BG-11 (*55*) with Turks Island Salt Mix 4X medium (*56*). We determined growth by measuring absorbance at 730 nm. For plates, we sterilized in an autoclave 250 ml of BG-11 medium and, once cooled, mixed it with a previously autoclaved solution containing 50 ml of TURK 4X concentration, 5 g of Bacto agar, and 275 ml of distilled H_2_O before pouring into petri dishes. If needed, we added kanamycin (25 μg/ml final concentration) just before pouring.

We used *E. coli* DH5α strain for cloning, maintenance, and propagation of plasmids constructed in this work (table S6). *E. coli* cells were grown in lysogeny broth (LB) medium overnight at 37°C with 200 rpm shaking. LB medium was prepared following the manufacturer’s instructions, and agar was added at a concentration of 15 g/liter for preparing LB plates. When needed, we added antibiotics to the medium at a final concentration of 50 μg/ml for ampicillin (Sigma-Aldrich) and 25 μg/ml for kanamycin (Sigma-Aldrich).

### Natural samples

For cyanobacteria detection in natural samples, we collected water (in triplicate) at about 2-m depth at the inner part of the estuarine system of Cádiz (36°29′ N, 6°13′ W) the next days: 7 March 2023 (two distinct locations) and 21 March 2023. Using 5 liters of freshly collected water, we performed screening for cyanobacteria by passing it through three consecutive meshes of 200, 100, and 20 μm. We analyzed several subsamples of 5 ml of the filtered water containing the phytoplankton size fraction of <20 μm using IFC, as described below.

### Generation of mutant strains of *Synechococcus* sp. PCC 7002

We constructed plasmid pRLi21a by cloning the gene encoding superfolder GFP (*sf-gfp*) between the Eco RI and Hind III sites of pTrc99A (*57*) followed by the insertion of the C.K1 kanamycin resistance cassette from pRL161 (*58*) in the Hind III site. We amplified by polymerase chain reaction (PCR) a DNA fragment containing neutral sites for recombination from the genomic DNA of *Synechococcus* sp. PCC 7002 using primer pairs NS1-LEFT-F/NS1-LEFT-R and NS1-RIGHT-F/NS1-RIGHT-R. We joined the fragments using the Gibson assembly protocol (*59*) to fragments from plasmid pRLi21a, which we amplified using the primer pairs pPC1-1F/pPC-1R and pPC-2F/pPC-1R. We named the resulting plasmid pPC-deliv2. We amplified the promoter of gene SYNPCC7002_A0740 encoding the adenosine 5′-triphosphate (ATP) synthase subunit I gene using primers Prom_A0740-A1 and Prom-A0740-A2 and the promoter of the gene SYNPCC7002_A1798 encoding the RuBisCO (ribulose-1,5-bisphosphate carboxylase/oxygenase) large subunit using primers Prom_rbc-A1 and Prom_rbc-A2. We respectively amplified the regions encoding the signal peptides of genes SYNPCC7002_A0500 and SYNPCC7002_A0313, both encoding periplasmic subunits of ATP-binding cassette (ABC) transporters, with primer pairs PepS_A0500-A2/PepS_A0500-A3 and PepS_A0500-A2/PepS_A0500-A2. We cloned all possible combinations of promoters and signal peptides using Gibson assembly upstream of the *sf-gfp* gene of plasmid pPC-deliv2 amplified with primers Vector-A1 and Vector-A3. We named the resulting plasmids pPC3, pPC4, pPC5, and pPC6. In these plasmids, sfGFP is expressed as a fusion protein carrying the corresponding signal peptide in its N terminus. The oligonucleotides and plasmids used in this work are shown in tables S6 and S7.

We transformed *Synechococcus* sp. PCC 7002 with the plasmids mentioned above using previous protocols (*60*, *61*) with modifications that included different concentrations of the plasmid DNA and cells. The culture was grown in BG-11 + TURK 4X (1:1 v/v) medium. We harvested cells by centrifugation (15,700*g* at 24°C), washed them twice with fresh medium, and resuspended them in fresh medium to obtain a final concentration of 30 to 40 μg of chlorophyll per milliliter (*62*).

We mixed this cell concentrate with 300 ng of plasmid DNA (<10-μl volume), previously extracted with WideUse Plasmid Purification Kit (Canvax), and incubated the mix for 5 hours at room temperature (RT). Then, we spread the transformation mixture on a sterile filter (Immobilon, 88-mm diameter, 0.45-μm pore, Millipore) positioned on BG-11 + TURK 4X concentration agar plates and, once dried, placed it in a culture chamber (30°C) with constant illumination (10 μE/m^2^ s) for 48 hours. Afterward, we transferred the filters to a plate with kanamycin (25 μg/ml) and refreshed them each week. We performed PCR of kanamycin-resistant colonies to confirm the insertions into the genome of *Synechococcus* sp. PCC 7002 using the primer pairs pPC-F and pPC-R. After PCR confirmation, we visualized the cells under the fluorescence microscope, as described below.

### Fluorescence microscopy

We used a Thunder Imager 3D Assay (Leica Microsystems) equipped with the GFP filter (excitation, 470/40; emission, 525/50) and Y5 filter (excitation, 620/60; emission, 700/75) with the 63× objective HC PL APO using immersion oil. To detect fluorescence from both sfGFP and calcein, we used the GFP filter, while we used the Y5 filter to detect cyanobacterial autofluorescence from the chlorophyll.

We observed samples stained with FM 1-43 with a Leica DM6000B fluorescence microscope equipped with ORCA-ER camera (Hamamatsu). We monitored fluorescence with a Tx2 filter (excitation, 560/40; emission, 645/75) for cyanobacterial autofluorescence or a fluorescein isothiocyanate (FITC) L5 filter (excitation band-pass, 480/40 nm; emission band-pass, 527/30 nm) for FM 1-43.

To prepare samples for the fluorescence microscope, we placed a silicone isolator (GBL664101-25EA, Grace Bio-Labs) on top of a glass slide (25 by 75 mm, 1.2 mm thick; EMS). We poured liquid BG-11 agar (1%) in each of the wells of the silicone isolator and let it solidify. Afterward, we placed 3 μl of the sample on top of the agar and let it dry before placing a high-precision microscope coverslip (24 by 50 mm, 1.5H; Marienfeld) on top and mounting the preparation on the fluorescence microscope for observation. The Advanced Optical Microscopy Unit of Instituto Maimónides de Investigación Biomédica de Córdoba (IMIBIC) assisted in the use of and training in fluorescence microscopy.

### Transmission electron microscopy

For TEM grid preparation, we harvested 100 ml of cell culture by centrifugation (8 min at 32,000*g* at RT), discarded the supernatant, resuspended in 1 ml of fixing solution [5:4:1 sodium cacodylate 0.2 M (pH 7):dH_2_O:25% glutaraldehyde grade I], and stored at 4°C until use. Afterward, on parafilm, we sequentially moved the grids (CF200-Cu, AGS160 Agar Scientific) over one drop of the fixed sample (10 μl, 5 min), two drops of water (40 μl, 1 min each), and two drops of 2% uranyl acetate in ethanol (20 μl, 1 min each); then, we gently blotted the excess liquid from the grid and let it dry.

To observe planktonic cells, we prepared the TEM grid as follows: We fixed 1 ml of culture in mid-exponential phase with 5 μl of 25% glutaraldehyde grade I for 1 hour and then performed the same protocol mentioned above by moving the grid sequentially over one drop of the fixed sample, two drops of water, and two drops of 2% uranyl acetate in ethanol. Specifically, for the image in fig. S4 (D and F), we grew 50 ml of axenic *Prochlorococcus* sp. MIT9313 cells to mid-exponential phase. Then, we placed 10 μl of well-mixed culture on a grid, which was immediately flash-frozen and prepared for imaging.

We also prepared TEM grids as follows: we harvested 250 ml of cell culture and resuspended it in 50 ml of fresh medium in a sterile Falcon tube. Afterward, we placed a grid floating in the culture and left it there for a week. Following this incubation, we fixed the culture, including the grid, with glutaraldehyde (final concentration 0.125%) for 1 hour. Then, we carefully removed the grid from the culture and sequentially moved it over two drops of water (40 μl, 1 min each) and two drops of 2% uranyl acetate in ethanol (20 μl, 1 min each) and gently blotted the excess liquid from the grid and let it dry. In parallel, we prepared another sample of the same culture in the same way but without the previous centrifugation (nonconcentrated sample).

To prepare the TEM grids in which we naturally promote cell adhesion, we used the same protocol mentioned above for the 50-ml cultures, leaving the grid to incubate for a week to observe nanotubes.

For ultrathin sections, we fixed the culture as described above, but after 24 hours, it was postfixed in 1% osmium tetroxide in 0.1 M cacodylate buffer for 1 hour. After washing in the same buffer, we transferred the pellet to a 1.5-ml Eppendorf tube where the embedding procedure was resumed. The pellet was then dehydrated in an ascendent series of ethanol (50, 70, and 90% and absolute ethanol) in steps of 20 min. We changed absolute ethanol three times (20 min each). Next, we treated the sample with propylene oxide and sequentially infiltrated in Embed 812 epoxy resin (EMS, USA). We used the sequence propylene oxide:resin 2:1, 1:1, and 1:2 for 24 hours (8 hours each). Afterward, we transferred the pellet to pure resin for 24 hours. The resin block was formed in the same Eppendorf tube with fresh resin at 65°C for 48 hours. We obtained thin sections (60 to 70 nm width) with an Ultracut Reicher ultramicrotome, mounted them on nickel grids, and stained with 2% uranyl acetate (in 50% ethanol) and lead citrate ready to use (EMS, USA).

We examined and photographed the different types of grids in a Jeol JEM 1400 transmission electron microscope at the Servicio Centralizado de Apoyo a la Investigación (SCAI; University of Córdoba, Spain) at 80 kV. We analyzed images and made measurements using the software ImageJ (https://imagej.net; National Institutes of Health, USA).

### Scanning electron microscopy

For SEM images (Fig. 1, A and B), the xenic *Prochlorococcus* SB culture, an HLII strain, was grown in a 25-ml Pro99 ESL culture under constant light at 75 μmol photons m^−2^ s^−1^ for approximately 1 week until the culture reached mid-exponential phase. During this period, we incubated a sterile 22 by 40 mm glass coverslip upright in the culture. Following this incubation, we fixed the sample, including the coverslip, with glutaraldehyde (final concentration of 0.125%, v/v) for 1 hour. Afterward, we dehydrated the coverslip with ethanol using a CD-3 critical point dryer (Tousimis 931 GL). We then sputter-coated the coverslip with gold particles using a HAR-053 EMS 150T S Metal Sputter Coater. We imaged the coverslip on an SEM-8 FESEM (field-emission scanning electron microscope) Ultra Plus. The Center for Nanoscale Systems at Harvard University assisted in the use of and training in SEM instrumentation and SEM sample preparation.

### Cell staining with fluorescent dyes

#### Calcein staining

We harvested 3 ml of exponentially grown cell culture and resuspended it in 1 ml of fresh growth medium then washed it twice with fresh medium and resuspended it in 0.5 ml of medium before adding 45 μl of calcein-AM [1 mg/ml in dimethyl sulfoxide (DMSO)] (Thermo Fisher Scientific). We incubated this suspension in the dark at RT for 3 hours, harvested the cells, and washed them five times with 1 ml of fresh medium. After the last wash, we resuspended the cells in 40 μl of medium to achieve a higher cell concentration for imaging. We incubated this suspension in the dark for 1 hour at RT before imaging with the fluorescence microscope (fig. S5). We used three separate controls for the calcein experiments. To ensure that only live cells acquire calcein fluorescence, we used cells boiled for 15 min as control (dead cells) in parallel with the living cells during the whole calcein staining protocol (figs. S16 and S17). Moreover, to ensure that there was no calcein left in the supernatant after the five washes, we added the supernatant from the last wash to a new tube with 3 ml of culture and incubated for 3 hours before imaging (fig. S6, E and F). The latest control we performed was to ensure that active exchange is needed. For that, we labeled two different cultures of *Synechococcus* sp. PCC 7002 cells with calcein; then, we boiled them for 15 min to kill the cells. Killed cells liberated hydrolyzed calcein-AM from the inside to outside of the cells after lysis. We incubated the supernatant of these two cultures of killed cells, which contain the hydrolyzed calcein-AM, with two different cultures of living cells with no calcein for 3 hours before imaging (fig. S7).

We observed calcein fluorescence at the fluorescence microscope using the GFP filter (excitation, 470/40 nm; emission, 525/50 nm) during 15 min of time lapse.

#### FM 1-43 staining

We added 2.5 μl of a solution of FM 1-43 (0.1 mg/ml; Molecular Probes) prepared in DMSO to 0.1 ml of culture at the exponential growth stage and incubated this mix for 10 min at RT. We washed the mix twice with fresh growth medium before observation at the fluorescence microscope with the FITC L5 filter (excitation band-pass, 480/40 nm; emission band-pass, 527/30 nm).

#### SYTOX Green staining

We stained samples using SYTOX Green dead cell stain (Invitrogen, Molecular Probes) at a final concentration of 30 nM and incubated them in the dark for 20 min at RT before observation at the Image Stream MKII with 488-nm excitation and 505- to 560-nm emission.

### Flow cytometry imaging

We took samples for flow cytometry throughout the calcein staining protocol to analyze calcein staining efficiency and cell viability. We analyzed both control and calcein-stained samples using a Becton Dickinson Accuri C6 Plus flow cytometer (BD Biosciences) equipped with a 488-nm laser. We selected populations by their chlorophyll fluorescence (FL3; emission, 670 nm longpass) and side scatter. Then, we represented calcein green fluorescence (FL1; emission, 533/30 nm) of these populations in a histogram and compared before and after the 3-hour incubation with calcein for both the control sample and the calcein-stained sample. We represented the data using the BD Accuri C6 Plus software (fig. S15).

### Imaging flow cytometry

We used an IFC with an ISX Amnis ImageStream^X^ Mark II (Luminex Corporation, Seattle, USA) to analyze samples after incubation with SYTOX Green. We collected these images at ×60 magnification at low flow rate and excited by a 488- and 785-nm laser using the INSPIRE software (Amnis Corp.). Chlorophyll autofluorescence was collected using 488-nm excitation and 642- to 745-nm emission; SYTOX Green fluorescence was collected using 488-nm excitation and 505- to 560-nm emission. A charge-coupled device camera collected images in the bright-field channel associated to each particle suspended in the analyzed sample. We performed post-acquisition spectral compensation and data analysis using the IDEAS 6.2 image analysis software package (Amnis Corp.). We gated cell densities of live, damaged, and dead cells in the dot plots of green fluorescence of SYTOX Green versus red autofluorescence of chlorophyll (Fig. 4).

We used SYTOX Green–stained *Synechococcus* sp. PCC 7002 cells analyzed with IFC to quantify the percentage of live and dead cells displaying nanotubes in our samples (see Supplementary Text).

We also used IFC to analyze natural samples. Chlorophyll autofluorescence (Ch05; 642- to 757-nm emission) and phycoerythrin autofluorescence (Ch03; 560- to 595-nm emission) were detected with a 488-nm excitation laser. Bright-field images were also acquired as described above, and post-acquisition spectral compensation and data analysis were performed using the IDEAS 6.2 image analysis software package (Amnis Corp.). We discerned cyanobacteria in the obtained cytograms by size (Ch06) and chlorophyll autofluorescence (Ch05) to then obtain the images of these cells.

### TEM and fluorescence image analysis

We processed TEM images using the software ImageJ (https://imagej.net) to measure the width and length of the nanotubes. For *Synechococcus* (sp. strains WH8102 and WH7803), we analyzed a total of 130 nanotubes (50 wide and 80 narrow). For *Prochlorococcus* sp. strain MIT9312, we analyzed a total of 95 nanotubes (26 wide and 69 narrow). For *Synechococcus* sp. strain PCC 7002, we analyzed a total of 65 nanotubes (18 wide and 45 narrow; see Supplementary Text).

We also used ImageJ to quantify fluorescence of the calcein experiments. We compared selected fields of images from the mixture of labeled and nonlabeled cells at *t* = 0 and *t* = 15 min. For that, we selected two different populations of cells (50 labeled and 50 nonlabeled cells) and measured them at each time point. We also measured the background fluorescence from cell-free regions and subtracted it from the fluorescence values obtained for each individual cell. We determined the fluorescence intensity of the two populations of cells within a field at each time point by averaging the fluorescence intensity from all individual cells previously selected (for the two populations separately). Then, we compared samples obtained at 0 and 15 min to see if the average fluorescence intensity of the nonlabeled cells increased over time. We did this procedure with three different combinations of cells: labeled *Synechococcus* sp. PCC 7002 + nonlabeled *Synechococcus* sp. PCC 7002 (combination 1), labeled *Synechococcus* sp. PCC 7002 + nonlabeled *Prochlorococcus* sp. MIT9313 (combination 2), and labeled *Prochlorococcus* sp. SS120 + nonlabeled *Synechococcus* sp. PCC 7002 (combination 3).

We studied the time-lapse analysis with Aivia software (version 12.1.0, LeiCa, Germany) as well. We segmented the cells, analyzed them using Cellpose and the Cell Analysis recipe, and further classified them by area and fluorescence intensity.

We selected 1172 cells for the calcein experiment of labeled *Prochlorococcus* sp. SS120 cells mixed with unlabeled *Synechococcus* sp. PCC 7002 (962 *Prochlorococcus* cells and 210 *Synechococcus* cells) and determined the time when they reached their maximum fluorescence (plotted in figs. S12 and S13).

### Identification of genes associated with the nanotube formation

We used BLASTP (*63*) to search genomes of marine *Prochlorococcus* and *Synechococcus* for homologs of the genes associated with the nanotube formation (tables S2 to S5). If no hits were found, we used DELTA-BLAST (*64*). Proteins used as queries are listed in table S2, and the following databases were inquired: *Synechococcus* sp. PCC 7002 (taxid:32049), *Synechococcus* sp. WH8102 (taxid:84588), *Prochlorococcus* sp. SB (taxid:59926), *Synechococcus* (taxid:1129), and *Prochlorococcus* (taxid:1218). We used default parameters and considered the hits with an *E* value of less than 1 × 10^−2^.

The two major cell wall hydrolases—LytC (*N*-acetylmuramoyl-l-alanine amidase) and its activator LytB—together are involved in removing the septal peptidoglycan and function in membrane migration during processes where the cell wall needs to be modified, such as cell division (*65*) and nanotube protrusion and connection with the recipient cell (*41*). Homologs of both *lytC* and *lytB* were identified in the genomes of *Synechococcus* and *Prochlorococcus* (tables S2 to S4). Specifically, ACA99878 protein in *Synechococcus* sp. PCC 7002 aligns to the SpoIID domain of LytB protein (table S3). The SpoIID domain is a lytic transglycosylase that degrades the glycan strands into disaccharide units but only after peptidoglycan stem peptides and peptide cross-bridges have been removed by the amidase (*65*) such as LytC. WP_254903423 in *Synechococcus* sp. PCC 7002 aligns to the *N*-acetylmuramoyl-l-alanine amidase domain of LytC (table S3). In general, two copies of *lytB*-like genes (also *spoIID*) and one to a few copies of *lytC*-like genes were identified in marine *Synechococcus* and *Prochlorococcus* genomes (tables S3 to S5).

The peptidoglycan endopeptidase LytE breaks the linkage of *m*-diaminopimelic acid of the peptidoglycan (*66*), and putative homologs are present in marine *Prochlorococcus* and *Synechococcus* (tables S3 to S5). In contrast, YmdB protein, which was shown to be important for nanotube formation in *B. subtilis* (*8*), was not found in picocyanobacteria. Future functional analysis will show if the putative homologs of *lytB*, *lytC*, and *lytE* found in picocyanobacterial genomes are involved in nanotube formation and what are the alternative genes that play in picocyanobacteria the function of the CORE genes in heterotrophic bacteria.
