# Supplementary material for: Direct interaction between marine cyanobacteria mediated by nanotubes
Source: Sci Adv. 2024 May 23;10(21):eadj1539. doi: 10.1126/sciadv.adj1539 (PMC11114229; doi:10.1126/sciadv.adj1539)
Supplement: Supplementary file 1 — Supplementary Text Figs. S1 to S19 Tables S1 to S7 [file sciadv.adj1539_sm.pdf]

Science Advances
AAAS

Supplementary Materials for

Direct interaction between marine cyanobacteria mediated by nanotubes

Elisa Angulo-Cánovas *et al.*

Corresponding author: María del Carmen Muñoz-Marín, b32mumam@uco.es;
José Manuel García-Fernández, jmgarcia@uco.es

Sci. Adv. **10**, eadj1539 (2024)
DOI: 10.1126/sciadv.adj1539

This PDF file includes:

Supplementary Text
Figs. S1 to S19
Tables S1 to S7

Supplementary Text

Nanotubes measurements

When measuring nanotubes from TEM images with ImageJ software (https://imagej.net. NIH; USA), the following data were obtained:

Synechococcus (sp. WH8102 and WH7803): wide nanotubes ranged in width from 108 to 200 nm ($\bar{x}$ = 156 nm; n = 50) and in length between 42 and 336 ($\bar{x}$ = 108 nm; n = 50); while narrow nanotubes ranged in width from 28 to 98 nm ($\bar{x}$ = 58 nm; n = 80) and in length between 32 and 498 nm ($\bar{x}$ = 151 nm; n = 80). The wide nanotubes of sp. PCC 7002 ranged in width from 110 to 185 nm ($\bar{x}$ = 133 nm; n = 18) and in length between 225 and 1.3 µm ($\bar{x}$ = 623 nm; n = 18); while narrow nanotubes ranged in width from 30 to 93 nm ($\bar{x}$ = 64 nm; n = 45) and in length between 93 and 1.7 µm ($\bar{x}$ = 771 nm; n = 45).

Prochlorococcus: wide nanotubes ranged in width from 105 to 199 nm ($\bar{x}$ = 160 nm; n = 26) and in length between 77 and 363 nm ($\bar{x}$ =170 nm; n = 26); while narrow nanotubes ranged in width from 18 to 98 nm ($\bar{x}$ = 53 nm; n = 69) and in length between 71 and 612 nm ($\bar{x}$ = 197 nm; n = 69).

When measuring nanotubes from flow cytometry images with ImageJ software, the following data were obtained:

Synechococcus (sp. PCC 7002): wide nanotubes ranged in width from 247 to 608 nm ($\bar{x}$ = 417 nm; n = 16) and in length between 1.1 and 2.4 µm ($\bar{x}$ = 2 µm; n = 16).

All micrographs from TEM were taken from 3 different grids. For each grid, 10 meshes of the grid (squares) were used to measure the total number of cells. In total we had at least 30 different meshes to measure and identify nanotubes in these cells.

Nanotubes count in live and dead cells

SYTOX Green-stained *Synechococcus* sp. PCC 7002 cells analyzed with IFC were used to quantify the percentage of live and dead cells displaying nanotubes in our samples. Cyanobacteria population was selected from the obtained cytograms from (Ch06 or particle size *vs* Ch05 or chlorophyll autofluorescence) by autofluorescence and size < 10 µm. Afterwards, this population was gated by positive signal of SYTOX Green to determine the population of dead cells. Once selected, nanotubes of the dead cell population were quantified. Subsequently, nanotubes were also quantified in cells with negative SYTOX Green signal (live cells). A total of 3,230 cells < 10µm were analyzed, of which 3,115 were alive and 115 were dying. A total of 186 nanotubes were identified (180 in live cells and 6 in dying cells) (Fig 4).

Nanotubes count in natural samples

A total of 282 cells of cyanobacteria were analyzed in triplicate on March 7, 2023, of which 5.7% ± 0.04 showed nanotubes. A total of 588 cyanobacterial cells were analyzed on the same day from another location, of which 4.3% ± 2.5 showed nanotubes. The last field sample collected on March 21, 2023 was also analyzed, showing 171 cyanobacterial cells of which 6.4 % showed nanotubes. In total, an average of 5.5 % ± 1.06 of cyanobacterial cells showed nanotubes in our field samples (Fig 5).

Nanotubes observation in concentrated and non-concentrated samples

We quantified the number of nanotubes using samples of two *Synechococcus* strains (WH8102 and WH7803) from a concentrated and non-concentrated culture by TEM. We used 25

TEM images for each condition (concentrated, non-concentrated) and strain, which were processed using ImageJ. For each image, the total number of cells and nanotubes was counted and then the ratio nanotubes/cell was calculated and used for the statistical analysis. Results are shown in Fig. S19.

For *Synechococcus* sp. WH8102: 54 nanotubes were observed in 331 cells in the non-concentrated samples, and 153 nanotubes in 1,297 cells in the concentrated samples. This resulted in an average of 0.164 nanotubes/cell (non-concentrated) vs 0.116 nanotubes/cell (concentrated). This difference is not significant (p value = 0.0668, n = 25), according to the t test.

For *Synechococcus* sp. WH7803: 68 nanotubes were observed in 473 cells in the non-concentrated culture, and 81 nanotubes in 824 cells concentrated culture. This resulted in an average of 0.155 nanotubes/cell (non-concentrated) vs 0.095 nanotubes/cell (concentrated) This difference is significant (p value 0.0026, n = 25). It is worth to notice that the average number of nanotubes was always lower in the concentrated samples than in the non-concentrated ones. These results show that nanotubes are produced naturally and therefore they are not an artifact derived from concentration methods.

<u>Time lapse analysis</u>

We plotted number of cells versus their maximum fluorescence at each frame of the 15 min-time lapse (fig S12 and S13) observing that in shorter times (0-2 min) most of the labeled *Prochlorococcus* cells (935 total cells: 729 near Pro and 206 far Pro) showed their maximum fluorescence. In contrast, most of the unlabeled *Synechococcus* cells (108 total cells: 73 near Syn and 35 far Syn) showed their maximum fluorescence in longer times (6.5 min) (fig S12 and S13). These data could explain that calcein is being transferred to *Synechococcus* unlabeled cells, since it takes them ~6.5 min to reach their maximum fluorescence during the time lapse.

<u>Calculation of surface-to-volume ratio</u>

We analyzed 10,000 particles of a *Synechococcus* sp. PCC 7002 culture to be able to determine the surface-to-volume ratio with IFC. From these particles, we counted 8531 live cells (Chl5 positive- Chla autofluoresce). Then, we discard non focused cells, and we selected images of focused cells in lateral view and separated from cells in frontal view obtaining 2196 images of PCC 7002 cells. We created a mask (morphological analysis of the image) for length (L) and width (W) measurements to distinguish the portion of the cell without nanotubes. The best geometric shape for the biovolume calculation of PCC 7002, seems to be a cylinder + 2 half spheres. However, the ISX does not provide data comparable to “height” in this formula since it measures the major and minor axis of the cell. Then we used the formula of prolate spheroid, where “height” would be the major axis (or length) and “diameter” the minor axis (or width) (*1*). We calculated for each cell its surface, volume and S/V ratio by using prolate spheroid formula. To calculate the S/V ratio of each cell with several nanotubes, we estimate the surface of one nanotube as a cylinder with ranges of width (0.1-0.2 µm) and length (2-5 µm) (Fig S19). Differences in cell S/V ratio with and without nanotube were tested by Kruskal-Wallis one-way ANOVA ($p<0,001$). When significant differences were detected, post-hoc pairwise comparison (Tukey’s tests) were performed between S/V cells without nanotube and each of the others (1, 2 and 3 nanotube). Significance levels were set up at $p<0,05$. All analysis were performed with Statistica 8.0 (StatSoft) program.

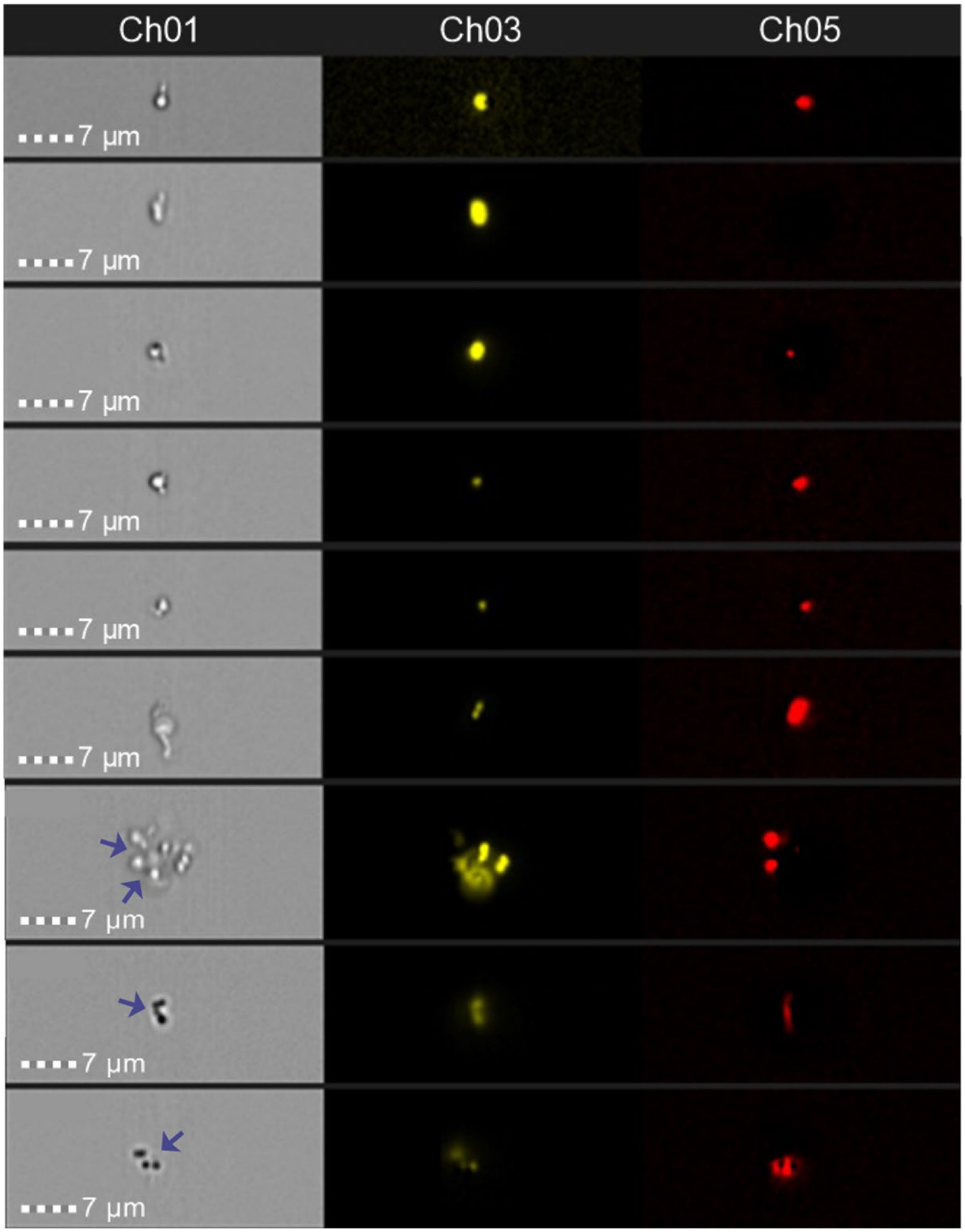

Figure S1. Nanotubes identified in *Synechococcus* sp. WH7803 cultures (solitary or into aggregates) by Imaging Flow Cytometry. Channel 1 (Ch01) shows brightfield images at 60X

magnification of *Synechococcus* sp. WH7803 cells with nanotubes, channel 3 (Ch03) shows phycoerythrin autofluorescence and channel 5 (Ch05) shows chlorophyll autofluorescence.

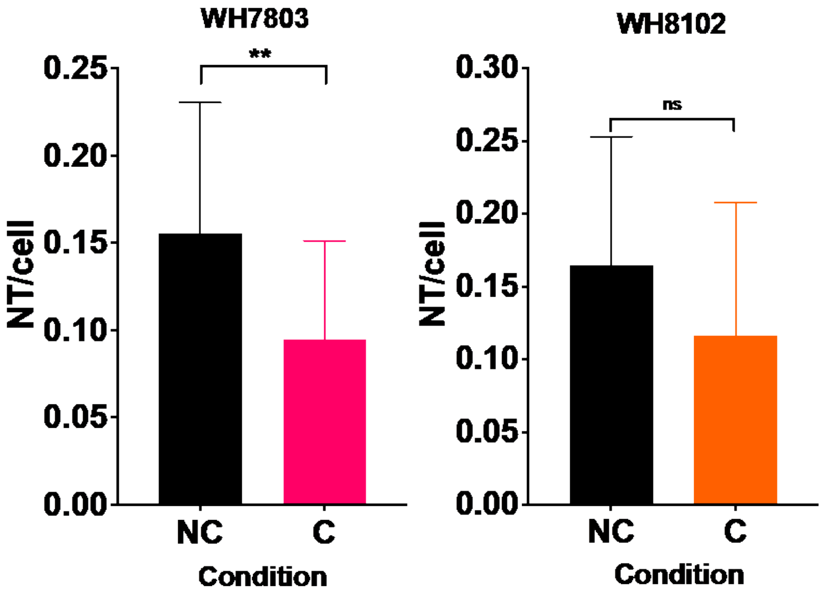

Figure S2. Total nanotubes/total cells observed per image (n = 25) in non-concentrated (NC) and concentrated (C) samples of *Synechococcus* sp. strains WH7803 and WH8102. Bars show the standard deviation. Further details are indicated in the text. Asterisks show the statistical significance of differences between non-concentrated and concentrated samples for each strain.

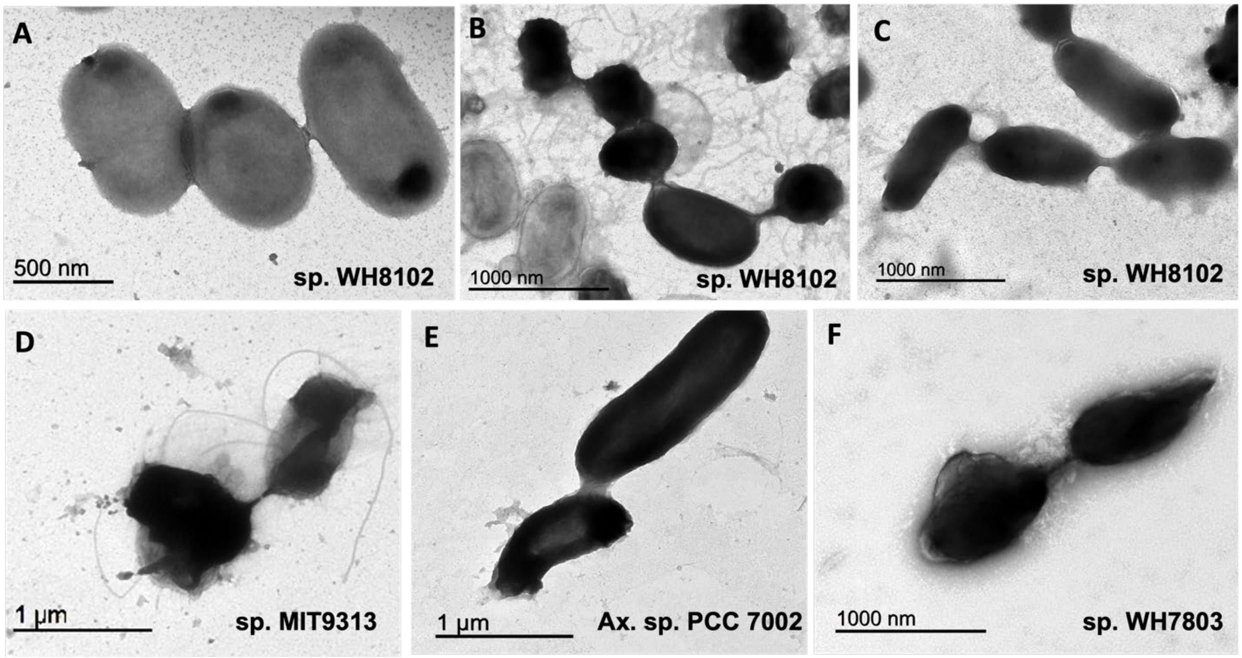

Figure S3. Transmission electron micrographs (TEM) of nanotubes formed between cyanobacterial cells grown to promote natural adherence. Micrographs of xenic *Synechococcus* sp. WH8102 **(A-C)**, xenic *Prochlorococcus* sp. MIT9313 **(D)**, axenic (Ax.) *Synechococcus* sp. PCC 7002 **(E)**, and xenic sp. WH7803 **(F)**.

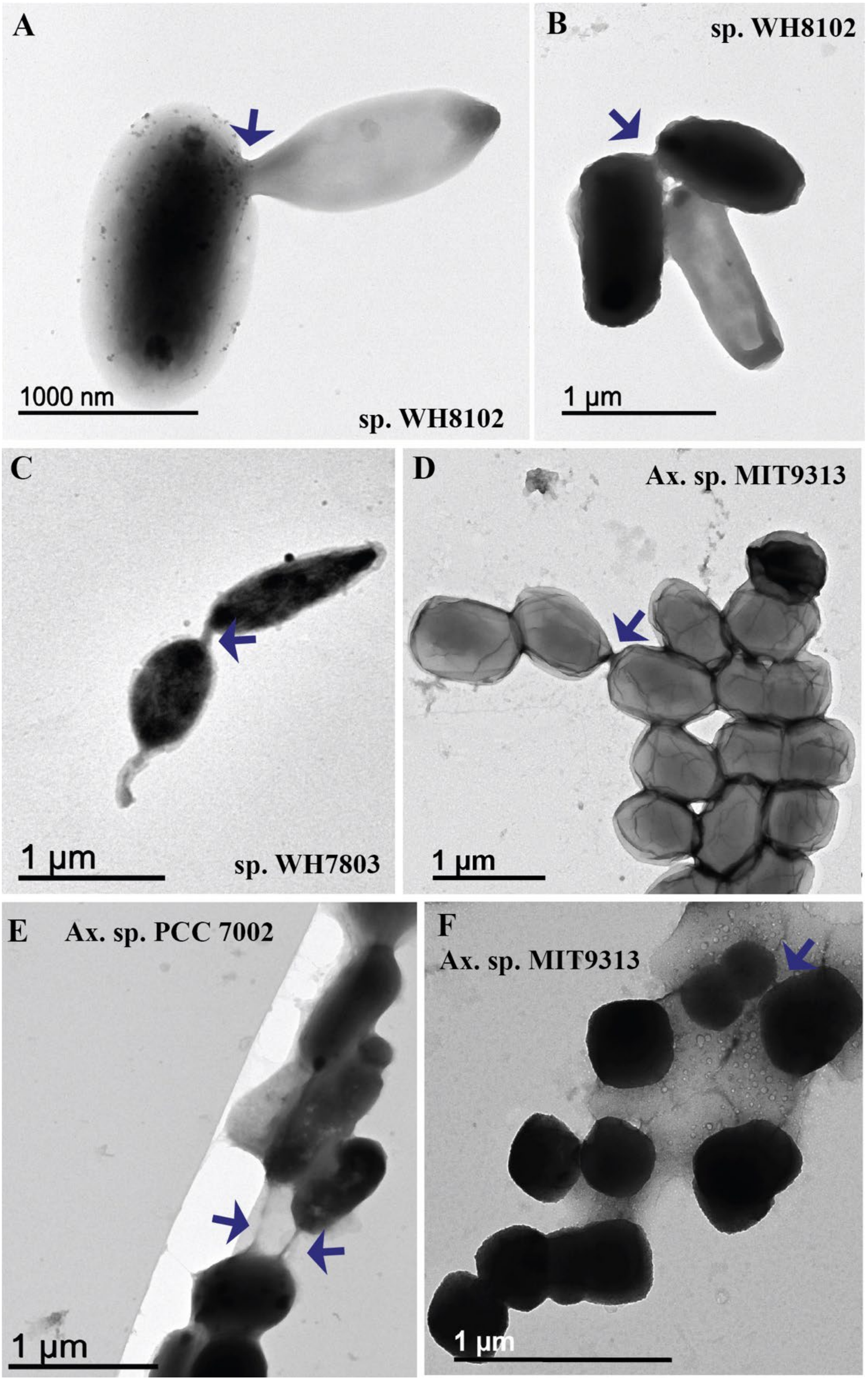

Figure S4. Transmission electron micrographs (TEM) of nanotubes formed between cyanobacterial planktonic cells. Micrographs of *Synechococcus* sp. WH8102 **(A, B)**, sp.

WH7803 **(B)**, axenic (Ax.) *Prochlorococcus* sp. MIT9313 **(D and F)**, and axenic *Synechococcus* sp. PCC 7002 **(E)**.

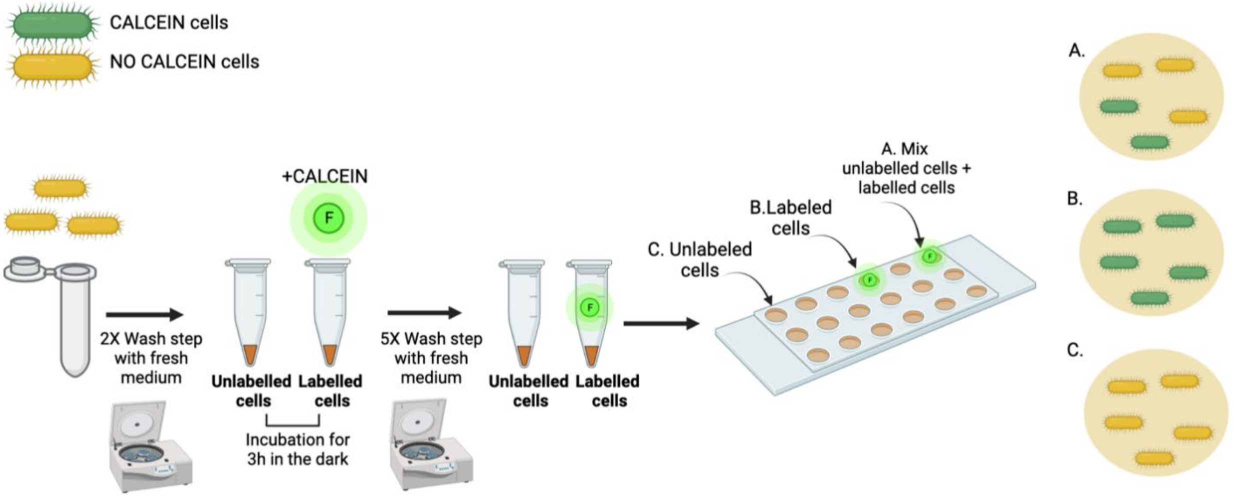

Figure S5. Schematic illustration of the experimental design for the calcein experiments.

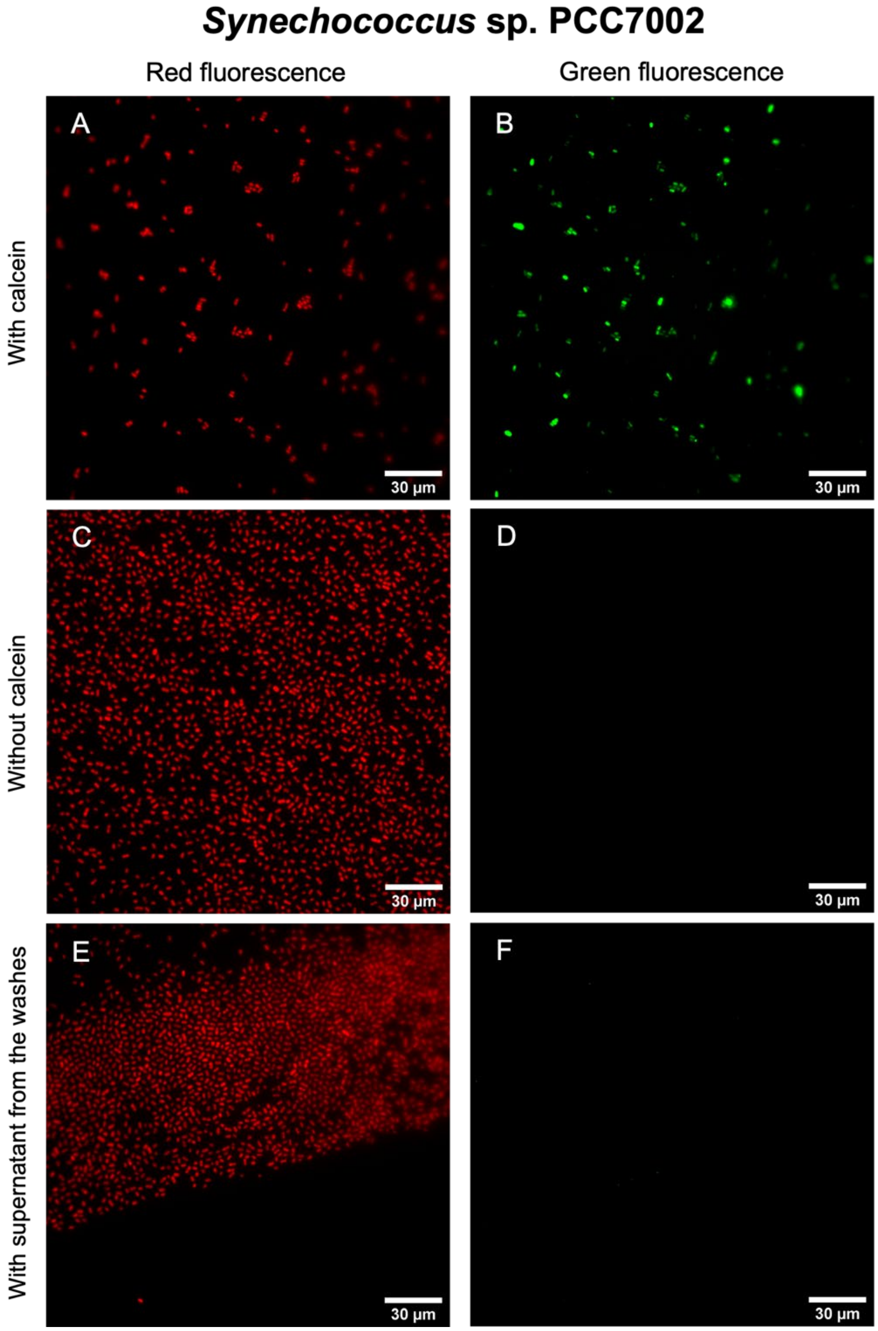

Figure S6. ***Synechococcus*** **sp. PCC 7002 cells are efficiently stained with Calcein-AM.** Fluorescence microscopy images of *Synechococcus* sp. PCC 7002 cells. Left column shows red chlorophyll fluorescence (autofluorescence) and right column shows green fluorescence (Calcein-AM). (A-B) Cells labeled with calcein. (C-D) Cells not labeled with calcein. (E-F) Cells incubated with the supernatant from the calcein-labeled cells.

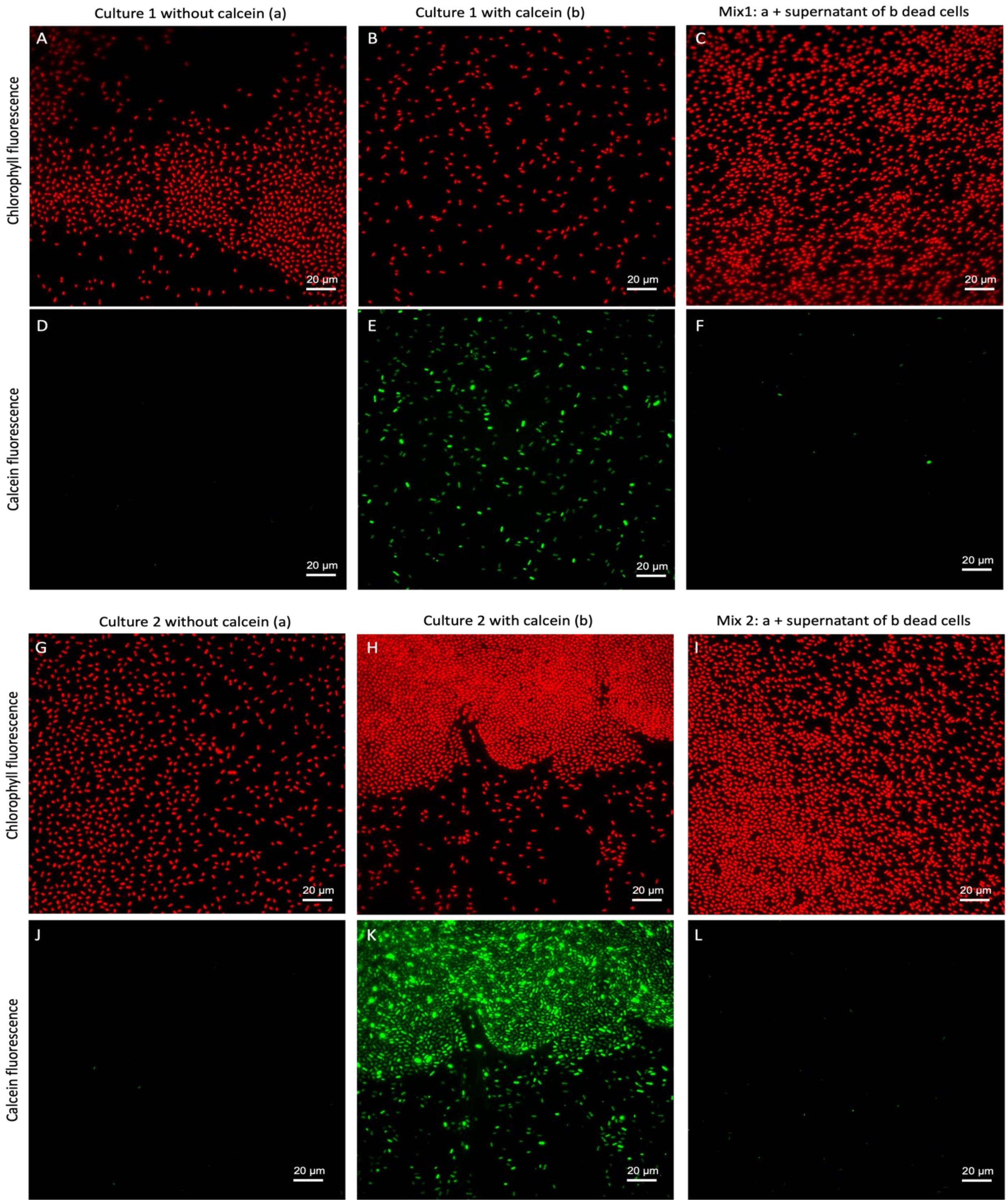

Figure S7. ***Synechococcus* sp. PCC 7002 cells are not stained with Calcein-AM coming from dead cells**. Fluorescence microscopy images of two cultures of *Synechococcus* sp. PCC 7002 cells. (A, D, G, J) Left column (a) shows cells not labeled with calcein. (B, E, H, K) Middle column (b) shows cells labeled with calcein. (C, F, I, L) Right column shows mix of cells not labeled with calcein with the supernatant obtained from dead cells labeled with calcein.

(A-C and G-I) show red chlorophyll fluorescence (autofluorescence) and (D-F and J-L) shows green fluorescence (Calcein-AM).

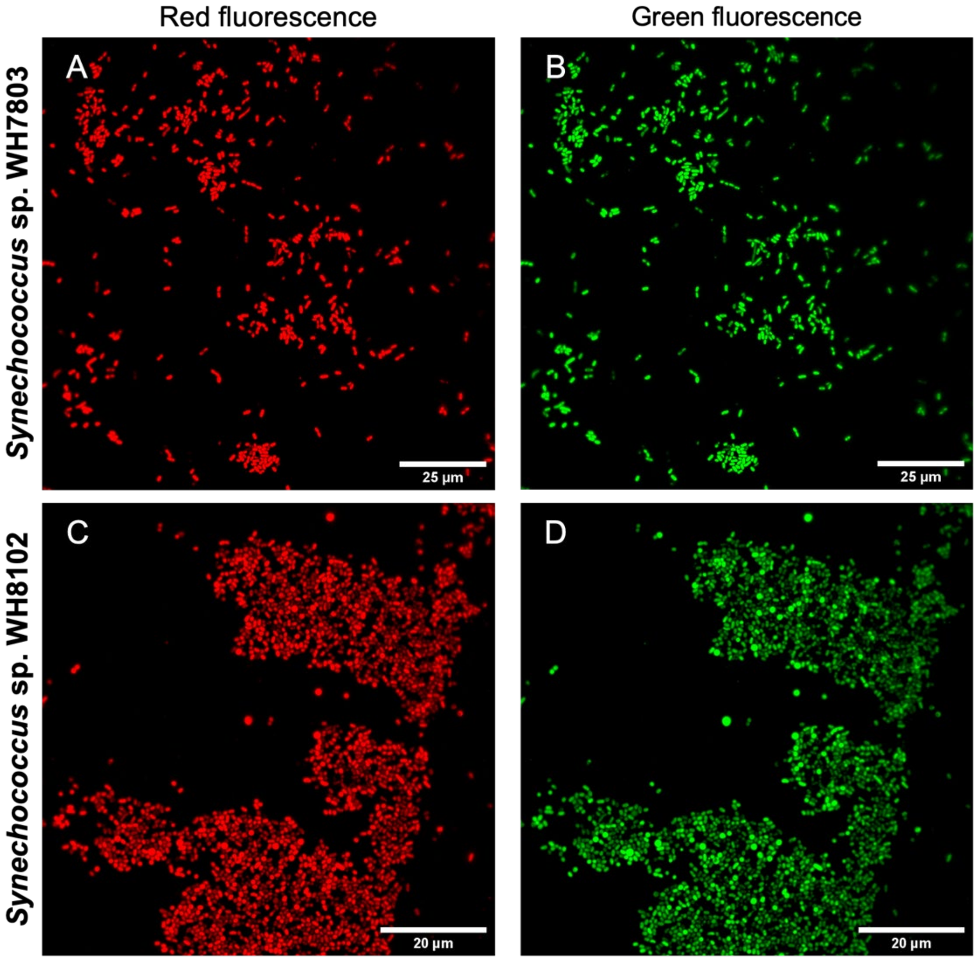

Figure S8. ***Synechococcus*** **sp. WH7803 (A and B) and WH8102 (C and D) cells without calcein addition showing autofluorescence in red and green channels.** The red channel (A and C) corresponds with chlorophyll and green (B and D) with phycoerythrin and phycocyanin pigments, the later overlapping with the calcein channel.

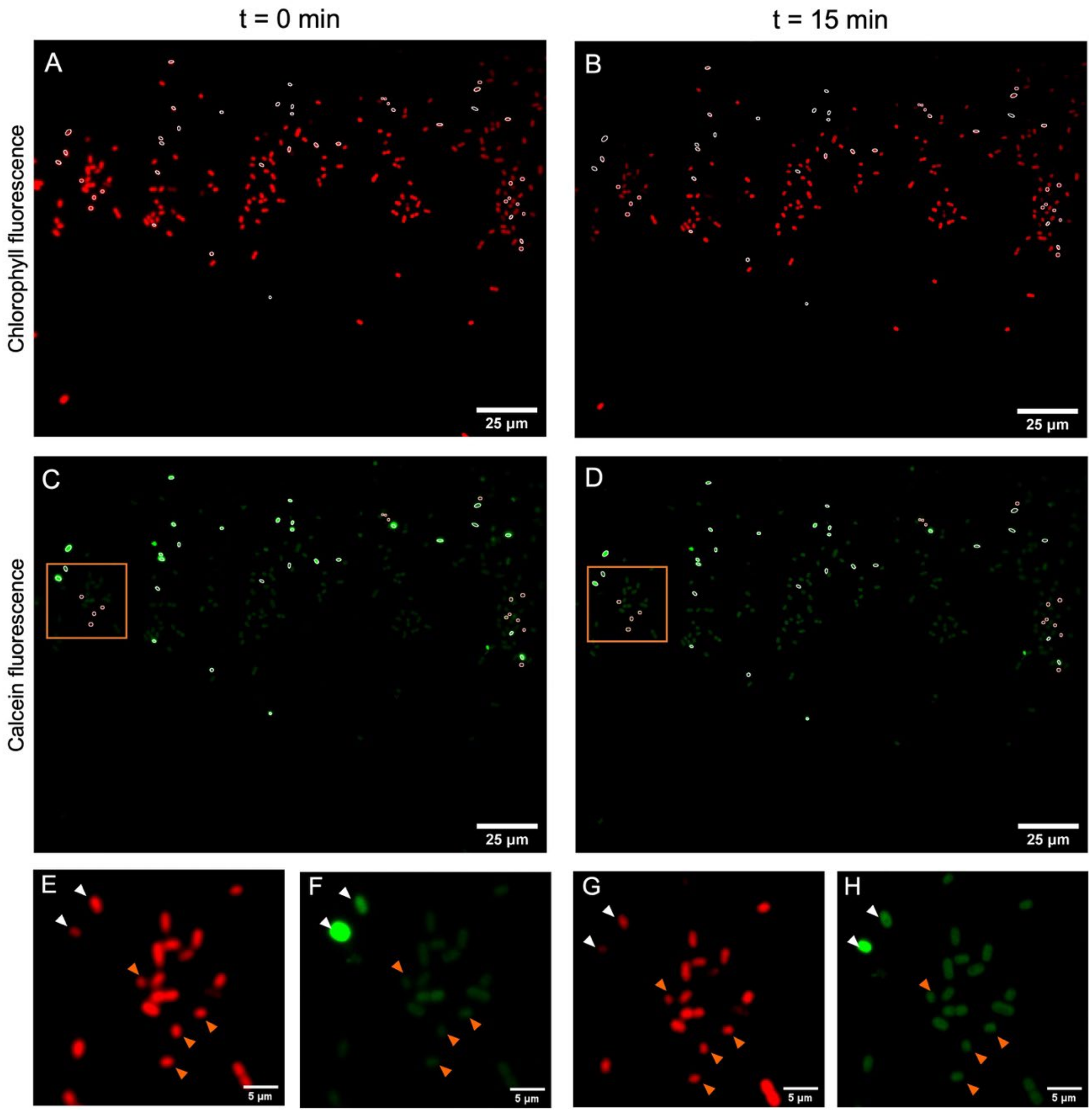

Figure S9. Transfer of calcein between *Synechococcus* sp. PCC 7002 and *Prochlorococcus* sp. MIT9313 cells. (A, B, C, D) *Synechococcus* sp. PCC 7002 calcein-labeled cells were mixed with unlabeled *Prochlorococcus* sp. MIT9313. 100 cells were selected: 50 calcein-labeled *Synechococcus* cells (white circles) and 50 unlabeled *Prochlorococcus* cells (pink circles). The average fluorescence intensity at t = 0 min *vs* t = 15 min (after the mix) was compared (table S1). **(E-H)** A magnification of the orange squares in C and D. White arrows highlight *Synechococcus* cells whose average fluorescence intensity decreases after 15 minutes. Orange arrows highlight *Prochlorococcus* cells whose average fluorescence intensity increases after 15 minutes. In panels E-G, *Synechococcus* cells appear with a size clearly bigger than *Prochlorococcus*.

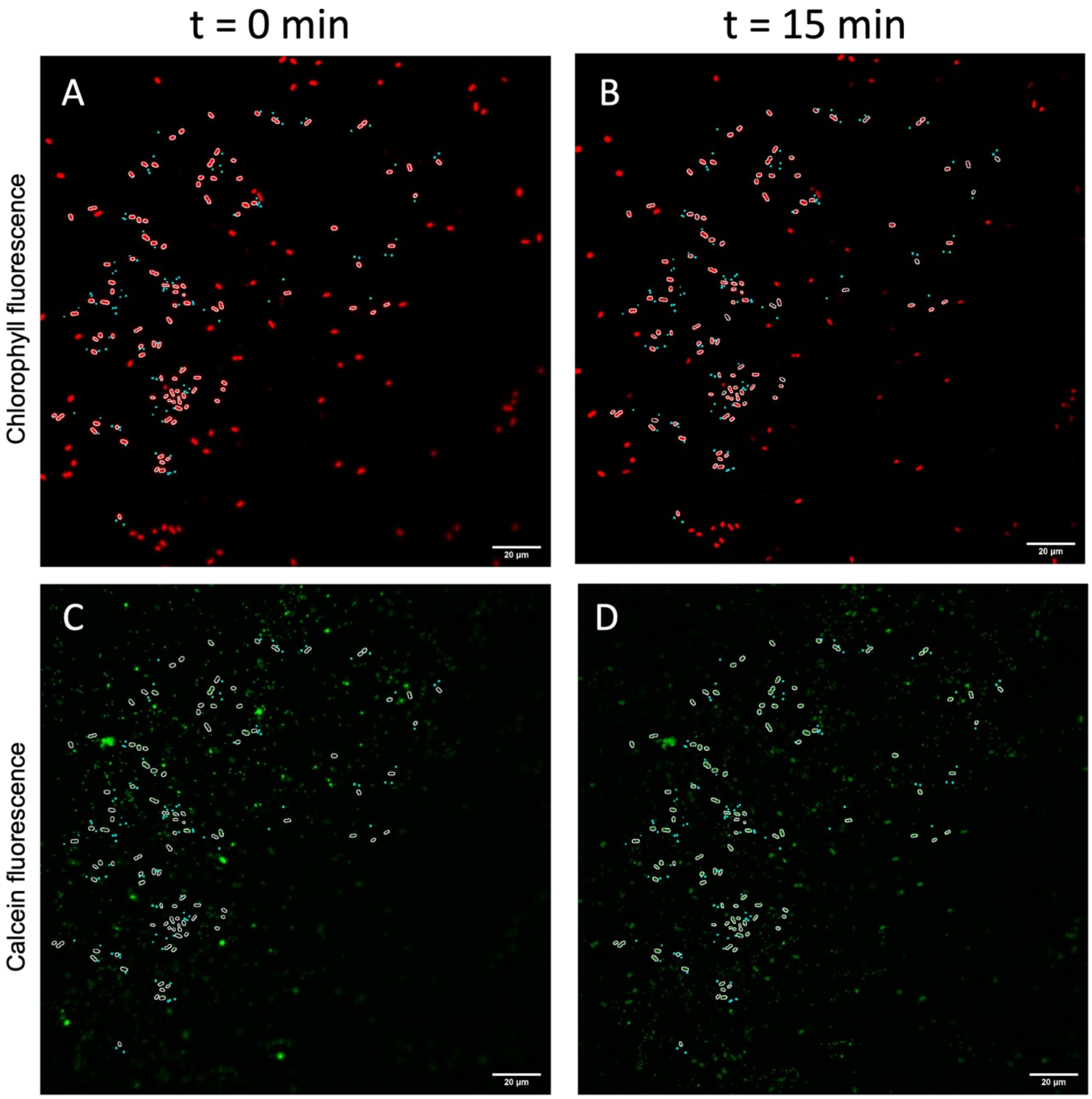

Figure S10. Transfer of calcein between *Prochlorococcus* sp. SS120 cells and *Synechococcus* sp. PCC 7002. *Prochlorococcus* sp. SS120 calcein-labeled cells were mixed with unlabeled *Synechococcus* sp. PCC 7002. 100 cells were selected: 50 calcein-labeled *Prochlorococcus* cells (cyan circle) and 50 unlabeled *Prochlorococcus* cells (white circle); and compared the average fluorescence intensity at t= 0 min (A, C) *vs* t = 15 min (B, D) (after the mix) (table S1). Top images correspond with the autofluorescence (chlorophyll) and below images with the immunofluorescence (calcein-AM).

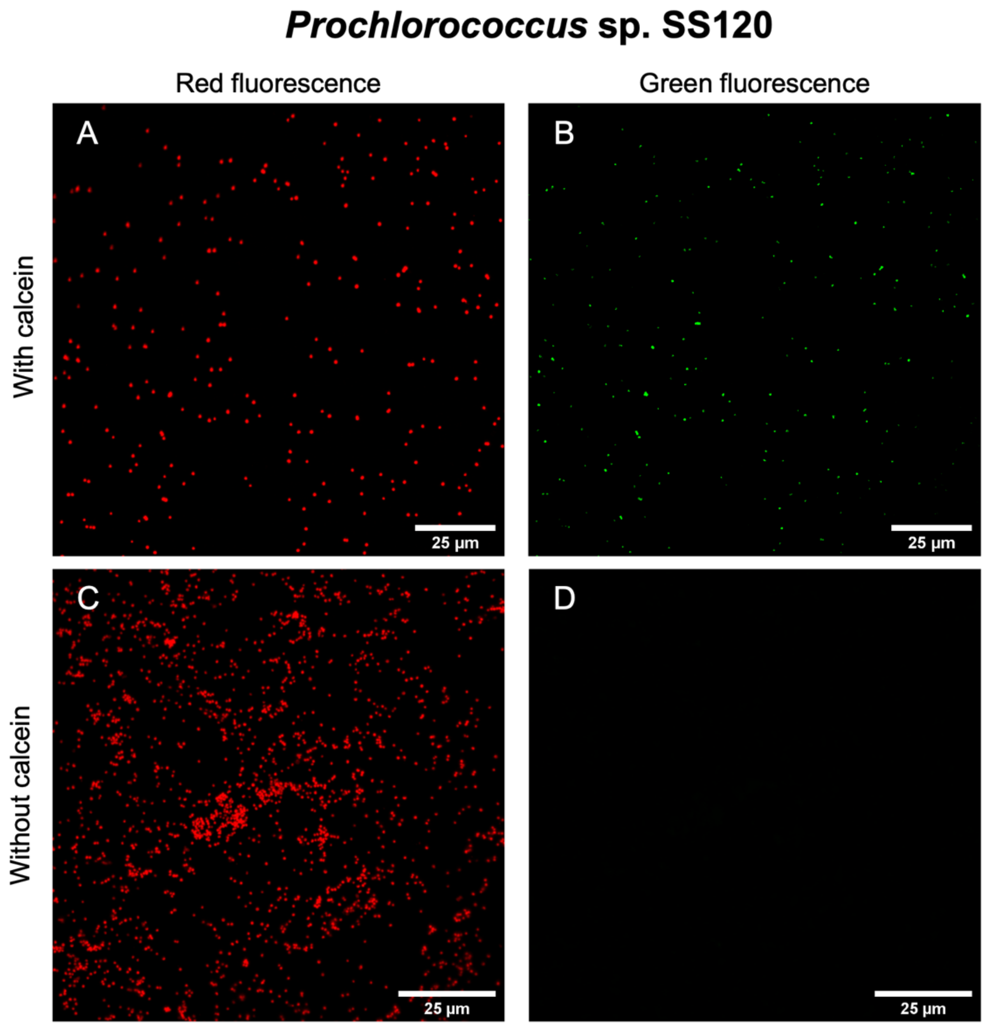

Figure S11. ***Prochlorococcus*** **cells are efficiently stained with Calcein-AM.** Fluorescence microscopy images of *Prochlorococcus* sp. SS120 cells. Left column shows red chlorophyll fluorescence (autofluorescence) and right column shows green fluorescence (Calcein-AM). (A-B) Cells labeled with calcein. (C-D) Cells not labeled with calcein.

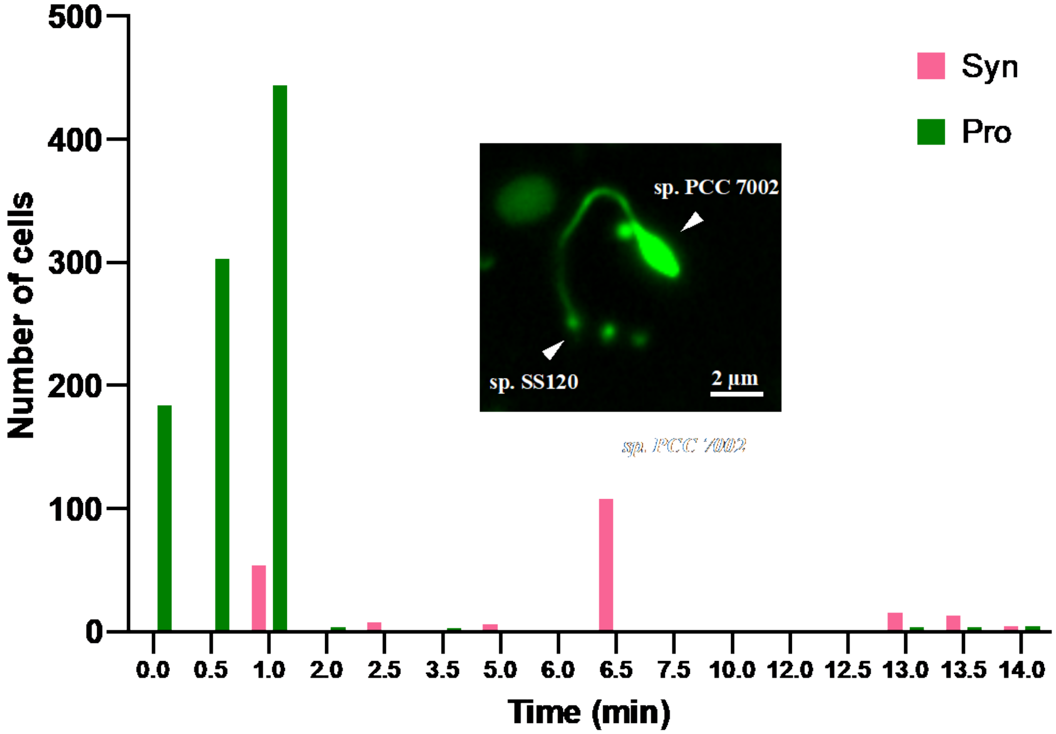

Figure S12. Number of cells with their maximum fluorescence in each time-lapse frame. Syn shows unlabeled *Synechococcus* sp. PCC 7002 cells and Pro labeled *Prochlorococcus* cells. Fluorescence microscopy image of *Prochlorococcus* sp. SS120 transferring calcein (green fluorescence) to the *Synechococcus* sp. PCC 7002 cell by the nanotube. A total of 1,172 cells were analyzed (962 *Prochlorococcus* cells and 210 *Synechococcus* cells).

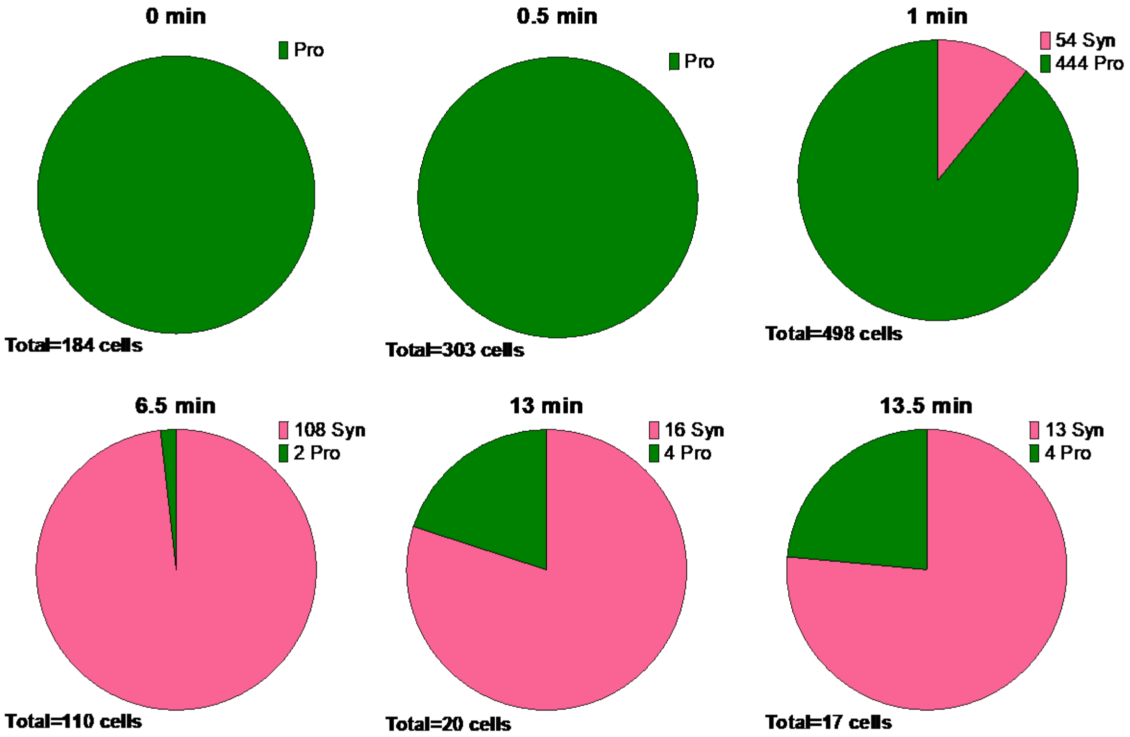

Figure S13. **Number of cells with their maximum fluorescence in 0, 0.5, 1, 6.5, 13 and 13.5 min**. Syn shows unlabeled *Synechococcus* sp. PCC 7002 cells and Pro shows the labeled *Prochlorococcus* sp. SS120 cells.

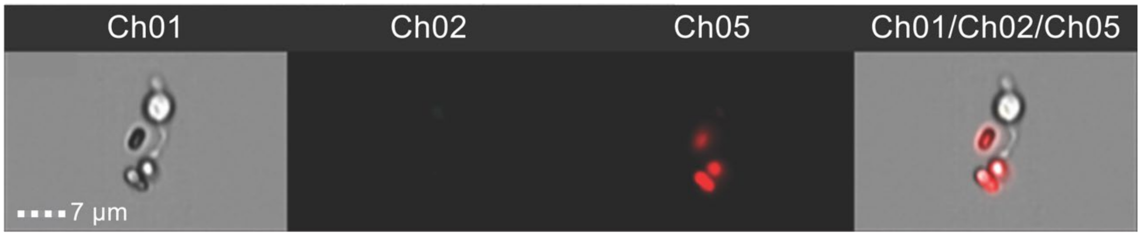

Figure S14. Interaction between *Synechococcus* sp. PCC7002 and other bacteria mediated by nanotubes in non-axenic culture of *Synechococcus* sp. PCC7002. Bright field images at 60x magnification of aggregates between *Synechococcus* sp. PCC7002 and heterotrophic bacteria observed by Imaging Flow Cytometry.

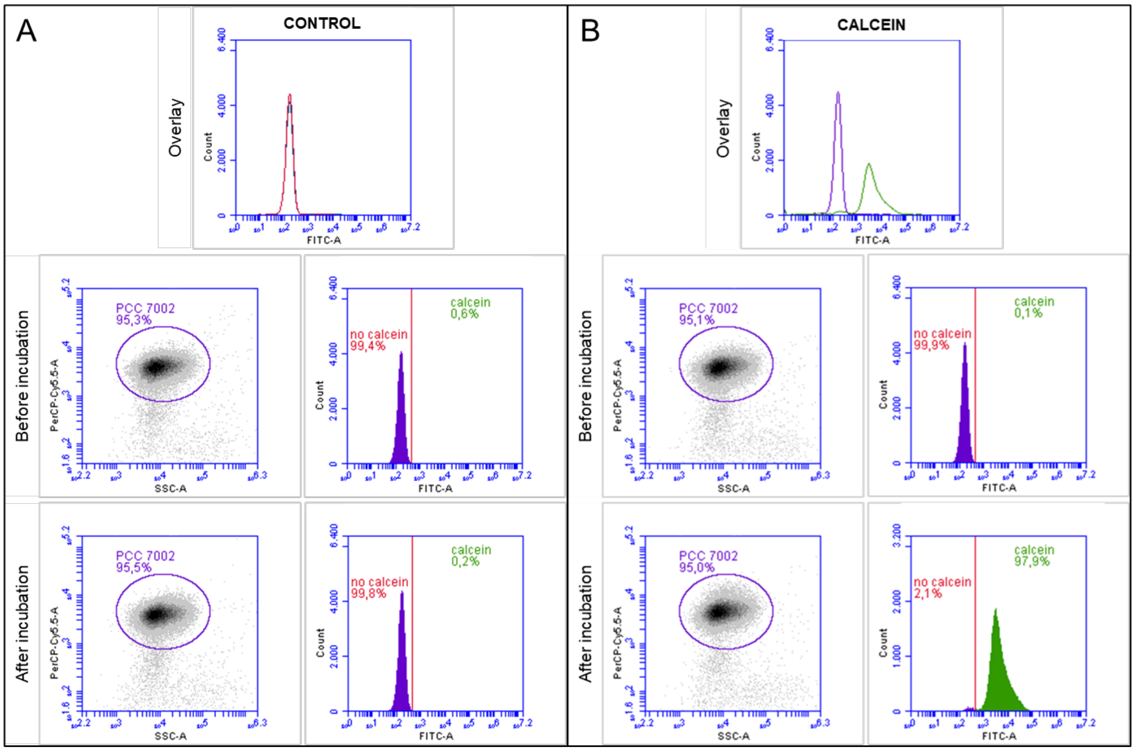

Figure S15. Monitoring Calcein-AM staining of *Synechococcus* sp. PCC 7002 by flow cytometry. Measurements were made before and after the 3-hour incubation period for both control (A) and calcein-added (B) sample. For each sample there is an overlay plot of the fluorescence before and after the incubation (first row); a density plot (FSC-A vs PerCP) showing chlorophyll fluorescence of the population and a histogram showing calcein fluorescence (FITC) gated by the population of *Synechococcus* sp. PCC 7002 (second and third rows).

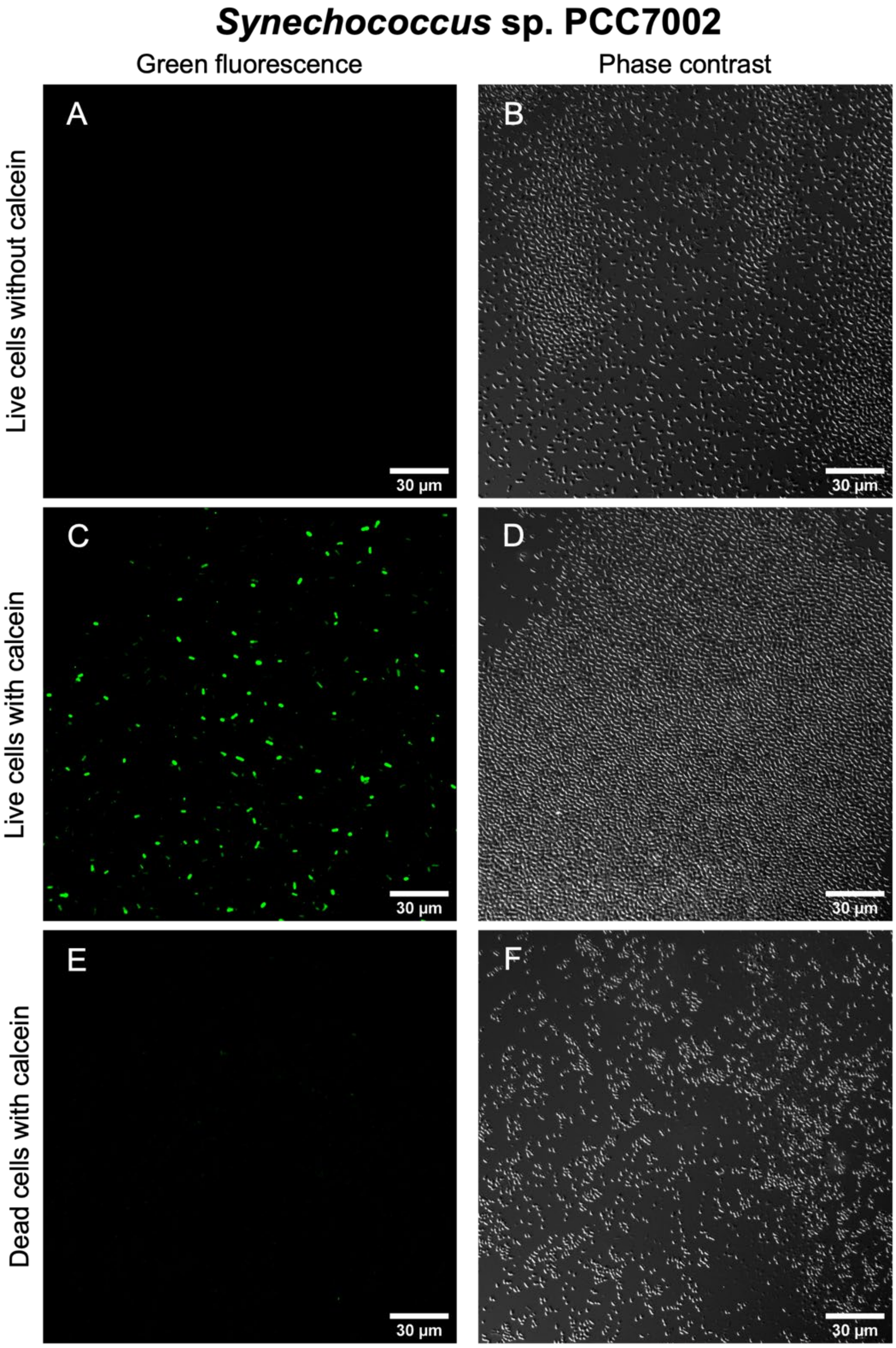

Figure S16. Fluorescence microscopy images of *Synechococcus* sp. PCC 7002 cells. Left column shows green fluorescence (calcein) images and right column shows phase contrast images. (A-B) Live cells without calcein. (C-D) Live cells labeled with calcein. (E-F) Dead cells labeled with calcein.

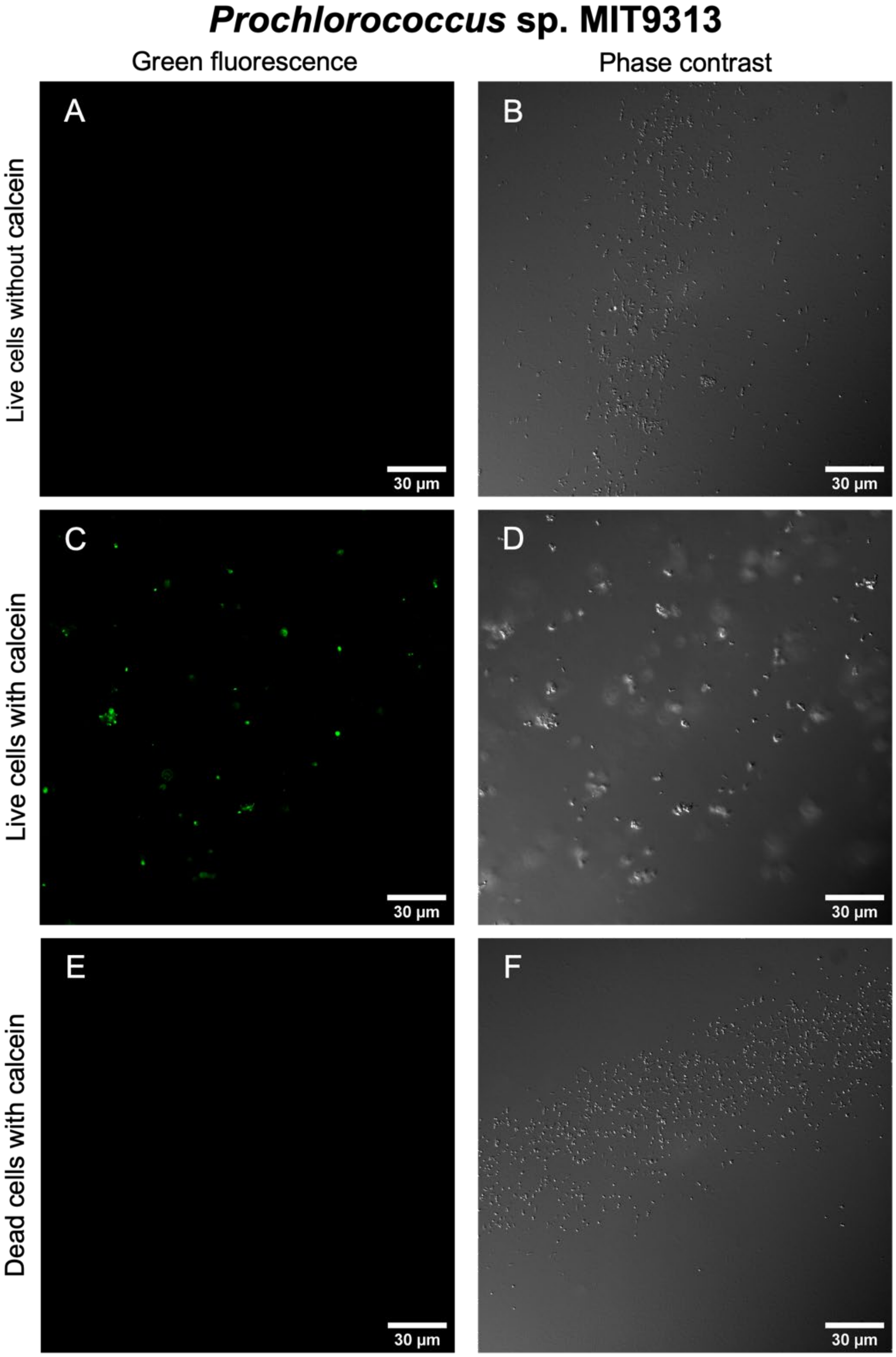

Figure S17. Fluorescence microscopy images of *Prochlorococcus* sp. MIT9313 cells. Left column shows green (calcein) fluorescence images and right column shows phase contrast images. (A-B) Live cells without calcein. (C-D) Live cells labeled with calcein. (E-F) Dead cells labeled with calcein.

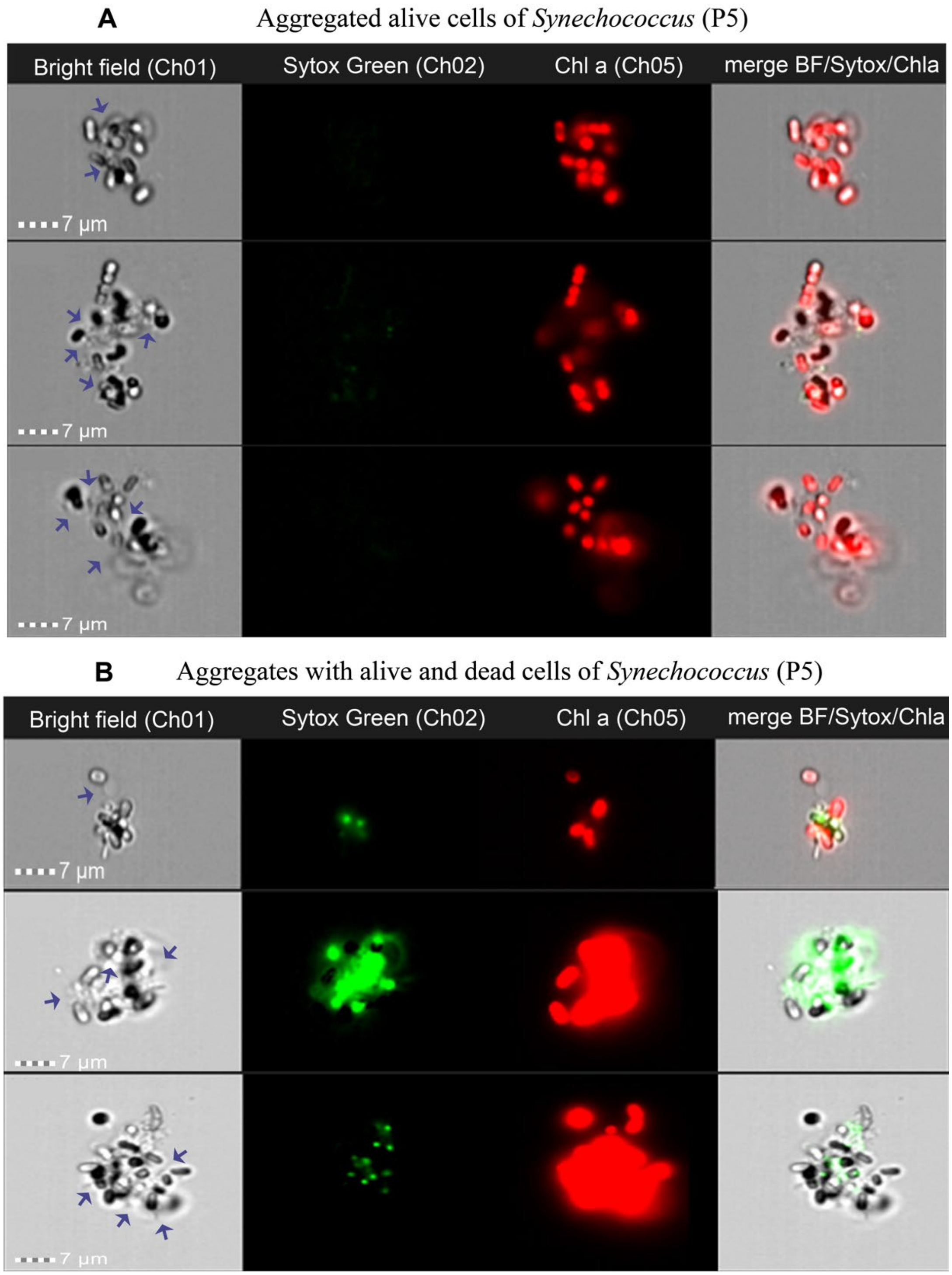

Figure S18. Correlation between nanotubes formation and vitality in subpopulation 5 of Figure 4 observed by IFC. Subpopulation P5 includes large size particles which are aggregates with alive *Synechococcus* cells (*Chl a* autofluorescence) **(A)** and aggregates with alive and dead *Synechococcus* cells (*Chl a* autofluorescence and SYTOX Green fluorescence **(B)**. Blue arrows show nanotubes in aggregated cells.

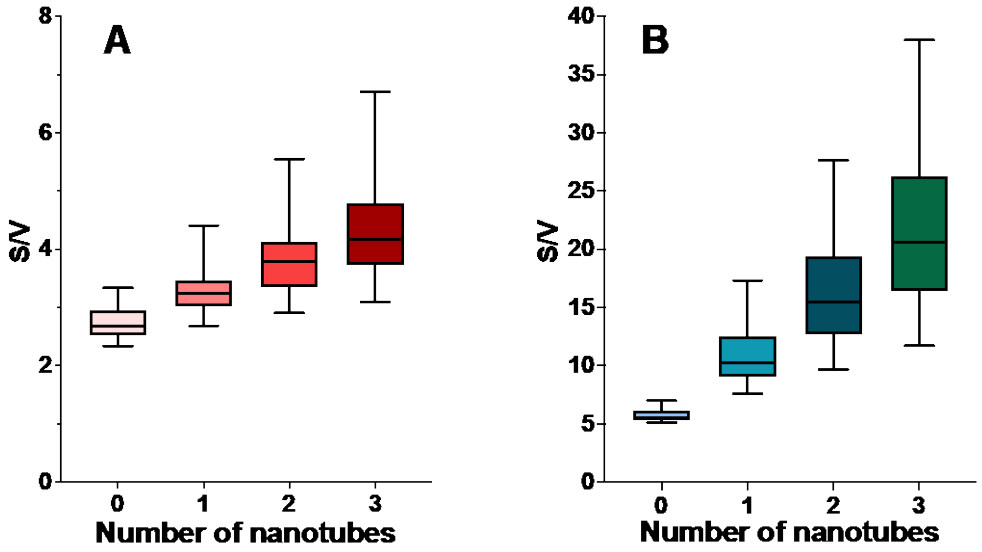

Figure S19. Box-plot for surface to volume ratios (S/V) calculated for individual cells without nanotubes (0 NT) or cells with one (1 NT), two (2 NT) or three nanotubes (3 NT). (A) large individual cells or two-three cells associations with an averaged (n =332) cell length of 4.20 ± 0.251 µm. and **(B)** small individual cells of an averaged (n =324) cell length of 2.06 ± 0.278 µm. We considered the surface of one nanotube as a cylinder with ranges of width (0.1-0.2 µm) and length (2-5 µm).

	Population	Average fluorescence intensity (a.u.) of the 50 cells – background fluorescence at t= 0 min	Average fluorescence intensity (a.u.) of the 50 cells – background fluorescence at t= 15 min	Δ over time (a.u)
**Combination 1** *Synechococcus* sp. PCC 7002 calcein-labeled cells mixed with *Synechococcus* sp. PCC 7002 non-labeled cells	Labeled cells	5384.6	1464.7	Decrease of 3191.9
	Non-labeled cells	539.2	734.4	Increase of 195.2
**Combination 2** *Synechococcus* sp. PCC 7002 calcein-labeled cells mixed with *Prochlorococcus* sp. MIT9313 non-labeled	Labeled cells	4697.9	2250.3	Decrease of 2447.6
	Non-labeled cells	256.04	331.55	Increase of 75.51
**Combination 3** *Prochlorococcus* sp. SS120 calcein-labeled cells mixed with *Prochlorococcus* sp. SS120 non-labeled cells	Labeled cells	769.97	148.85	Decrease of 621.13
	Non-labeled cells	218.3	251.3	Increase of 33.08

Table S1. Average fluorescence intensity in arbitrary units (a.u.) of the three combinations of cells used at t = 0 min and t = 15 min and difference between timepoints of the two cell populations (labeled and non-labeled with calcein-AM).

Functional group	Protein	NCBI ID	Protein annotation	Organism
CORE	FliP	NP_389517.1	flagellar biosynthesis protein FliP	*Bacillus subtilis* subsp. subtilis str. 168
CORE	FliQ	NP_389518.1	flagellar type III secretion system protein FliQ	*Bacillus subtilis* subsp. subtilis str. 168
CORE	FliR	NP_389519.2	flagellar type III secretion system protein FliR	*Bacillus subtilis* subsp. subtilis str. 168
CORE	FlhA	NP_389521.2	flagellar type III secretion system protein FlhA	*Bacillus subtilis* subsp. subtilis str. 168
CORE	FlhB	NP_389520.1	flagellar type III secretion system protein FlhB	*Bacillus subtilis* subsp. subtilis str. 168
CORE	EscR	NP_312610.2	T3SS structure protein EscR	*Escherichia coli* O157:H7 str. Sakai
CORE	EscS	NP_312609.1	type III secretory pathway, component EscS	*Escherichia coli* O157:H7 str. Sakai
CORE	EscT	NP_312608.1	type III secretion protein SpaR/YscT/HrcT	*Escherichia coli* O157:H7 str. Sakai
CORE	EscU	NP_312607.1	EscU/YscU/HrcU family type III secretion system export apparatus switch protein	*Escherichia coli* O157:H7 str. Sakai
CORE	EscV	NP_312596.1	type III secretory pathway, component EscV	*Escherichia coli* O157:H7 str. Sakai
Cell wall hydrolysis	LytE	NP_388823.2	cell wall dl-endopeptidase; phosphatase-associated protein (major autolysin)	*Bacillus subtilis* subsp. subtilis str. 168
Cell wall hydrolysis	LytB	NP_391443.1	putative cell wall-binding domain [Cell wall/membrane/envelope biogenesis]	*Bacillus subtilis* subsp. subtilis str. 168
Cell wall hydrolysis	LytC	NP_391442.1	N-acetylmuramoyl-L-alanine amidase (major autolysin)	*Bacillus subtilis* subsp. subtilis str. 168
Regulation	YmdB	WP_003245138	YmdB, a global regulator of late adaptive responses; a phosphodiesterase	*Bacillus*: Multispecies

Table S2. Description of proteins involved in nanotube formation.

Protein	Query ID	Subject ID	Percent identity to query	Query alignment coverage	E value	Notes
FliP	NP_389517.1	NA	NA	NA	NA	delta-blast no hits
FliQ	NP_389518.1	NA	NA	NA	NA	delta-blast no hits
FliR	NP_389519.2	NA	NA	NA	NA	delta-blast no hits
FlhA	NP_389521.2	NA	NA	NA	NA	delta-blast no hits
FlhB	NP_389520.1	NA	NA	NA	NA	delta-blast no hits
EscR	NP_312610.2	NA	NA	NA	NA	delta-blast no hits
EscS	NP_312609.1	NA	NA	NA	NA	delta-blast no hits
EscT	NP_312608.1	NA	NA	NA	NA	delta-blast no hits
EscU	NP_312607.1	NA	NA	NA	NA	delta-blast no hits
EscV	NP_312596.1	NA	NA	NA	NA	delta-blast no hits
LytE	NP_388823.2	WP_012308356.1	25.9	29	1.00E-20	LysM peptidoglycan-binding domain-containing M23 family metallopeptidase; confirmed with Delta-BLAST
LytE	NP_388823.2	WP_012307784.1	30	14	7.00E-03	C40 family peptidase; pfam00877
LytB	NP_391443.1	ACA99878.1	31.3	40	1.00E-29	SpoIID/LytB domain-containing protein; aligns to SpoIID domain only
LytB	NP_391443.1	WP_012307251.1	31.6	40	1.00E-19	SpoIID/LytB domain-containing protein; aligns to SpoIID domain only
LytC	NP_391442.1	WP_012306209.1	46	34	2.00E-38	N-acetylmuramoyl-L-alanine amidase; does not have LyB
LytC	NP_391442.1	WP_012306215.1	31.2	36	1.00E-17	N-acetylmuramoyl-L-alanine amidase; does not have LyB
YmdB	WP_003245138	NA	NA	NA	NA	delta-blast no hits

Table S3. Protein hits in *Synechococcus* PCC7002 (taxid:32049)

Protein	Query ID	Subject ID	Percent identity to query	Query alignment coverage	E value	Notes
FliP	NP_389517.1	NA	NA	NA	NA	delta-blast no hits
FliQ	NP_389518.1	NA	NA	NA	NA	delta-blast no hits
FliR	NP_389519.2	NA	NA	NA	NA	delta-blast no hits
FlhA	NP_389521.2	NA	NA	NA	NA	delta-blast no hits
FlhB	NP_389520.1	NA	NA	NA	NA	delta-blast no hits
EscR	NP_312610.2	NA	NA	NA	NA	delta-blast no hits
EscS	NP_312609.1	NA	NA	NA	NA	delta-blast no hits
EscT	NP_312608.1	NA	NA	NA	NA	delta-blast no hits
EscU	NP_312607.1	NA	NA	NA	NA	delta-blast no hits
EscV	NP_312596.1	NA	NA	NA	NA	delta-blast no hits
LytE	NP_388823.2	WP_011128815.1	28.9	36	2.00E-12	LysM peptidoglycan-binding domain-containing protein
LytE	NP_388823.2	WP_011128971.1	24.8	27	5.00E-04	C40 family peptidase
LytB	NP_391443.1	WP_083810650.1	30.2	45	2.00E-26	SpoIID/LytB domain-containing protein; aligns to SpoIID domain only
LytB	NP_391443.1	WP_011126937.1	36.4	16	3.00E-15	SpoIID/LytB domain-containing protein; aligns to SpoIID domain only
LytB	NP_391443.1	CAE07054.1	28.7	56	2.00E-26	similar to stage II sporulation protein D
LytC	NP_391442.1	WP_225867742.1	39.4	35	9.00E-31	N-acetylmuramoyl-L-alanine amidase
LytC	NP_391442.1	CAE07524.1	39.4	35	3.94E+01	N-acetylmuramoyl-L-alanine amidase
YmdB	WP_003245138	NA	NA	NA	NA	a weak hit to a hypothetical protein with E value=0.044; SYNW0778

Table S4. Protein hits in *Synechococcus* WH8102 (taxid:84588)

Protein	Query ID	Subject ID	Percent identity to query	Query alignment coverage	E value	Notes
FliP	NP_389517.1	NA	NA	NA	NA	delta-blast no hits
FliQ	NP_389518.1	NA	NA	NA	NA	delta-blast no hits
FliR	NP_389519.2	NA	NA	NA	NA	delta-blast no hits
FlhA	NP_389521.2	NA	NA	NA	NA	delta-blast no hits
FlhB	NP_389520.1	NA	NA	NA	NA	delta-blast no hits
EscR	NP_312610.2	NA	NA	NA	NA	delta-blast no hits
EscS	NP_312609.1	NA	NA	NA	NA	delta-blast no hits
EscT	NP_312608.1	NA	NA	NA	NA	delta-blast no hits
EscU	NP_312607.1	NA	NA	NA	NA	delta-blast no hits
EscV	NP_312596.1	NA	NA	NA	NA	delta-blast no hits
LytE	NP_388823.2	WP_032519589.1	30.4	36	2.00E-06	LysM peptidoglycan-binding domain-containing protein
LytE	NP_388823.2	WP_032520089.1	31.7	26	3.00E-06	C40 family peptidase
LytB	NP_391443.1	WP_052043492.1	28.4	44	9.00E-29	SpoIID/LytB domain-containing protein; aligns to SpoIID domain only
LytB	NP_391443.1	WP_032519945.1	25.8	41	4.00E-12	SpoIID/LytB domain-containing protein; aligns to SpoIID domain only
LytC	NP_391442.1	WP_032519270.1	42.3	36	6.00E-34	N-acetylmuramoyl-L-alanine amidase
YmdB	WP_003245138	NA	NA	NA	NA	NA

Table S5. Protein hits in *Prochlorococcus* SB (taxid:59926)

NAME	DESCRIPTION	COMMENTS
pRLi21a	A derivative of pTrc99A carrying the *sf-gfp* gene and the C.K1 kanamycin resistance cassette	Used as platform for further constructions
pPC-deliv2	A derivative of pRLi21a carrying the *trc* promoter upstream of the superfolder-GFP gene, a kanamycin resistance cassette and the *rrnB* terminator from *E. coli* flanked by sequences NS1-LEFT and NS1-RIGHT for recombination in a neutral site of the *Synechococcus* sp. PCC 7002 genome.	Expresses sf-GFP in the cytoplasm of *Synechococcus*
pPC3	A derivative of pPC-deliv2 where the *trc* promoter was replaced by the promoter of the RuBisCO large subunit gene and the signal peptide of the GN=SYNPCC7002_A0313 gene upstream of the *sf-gfp* gene	Expresses sf-GFP in the periplasm of *Synechococcus*
pPC4	A derivative of pPC-deliv2 where the *trc* promoter was replaced by promoter of the RuBisCO large subunit gene and the signal peptide of the GN=SYNPCC7002_A0500 gene upstream of the *sf-gfp* gene	Expresses sf-GFP in the periplasm of *Synechococcus*
pPC5	A derivative of pPC-deliv2 where the *trc* promoter was replaced by promoter of the GN=SYNPCC7002_A0740 gene and the signal	Expresses sf-GFP in the periplasm of *Synechococcus*

	peptide of the GN=SYNPCC7002_A0313 gene upstream of the *sf-gfp* gene	
pPC6	A derivative of pPC-deliv2 where the *trc* promoter was replaced by promoter of the GN=SYNPCC7002_A0740 gene and the signal peptide of the GN=SYNPCC7002_A0500 gene upstream of the *sf-gfp* gene	Expresses sf-GFP in the periplasm of *Synechococcus*

Table S6. Plasmids constructed for the generation of mutant strains of *Synechococcus* sp. PCC 7002.

NAME	SEQUENCE
NS1-LEFT-F	gcatgcatCAAGCCCAAGGGTTTCGTAGG
NS1-LEFT-R	cgttgcgcAGATCGAAAGAAAGAGGATCC
NS1-RIGHT-F	ctttttgcAGCTTCATTATATAGAATTTC
NS1-RIGHT-R	gtagaaacTTGCGAATGTGATTGTAGAAG
pPC1-1F	TTCGATCTgcgcaacgcaattaatgtgag
pPC1-1R	TGGGCTTGatgcatgccgcttcgccttcg
pPC1-2F	ATTCGCAAgtttctacaaactctttttgttta
pPC1-2R	ATGAAGCTgcaaaaaggccatccgtcagg
Prom_A0740-A1	TCGGGGGCCCCCGGGGGATAATGGCGTGATATGATTGCACGTG
Prom-A0740-A2	TCGGGCCCtcctCCGGGGATTGGGGTGTTACTTGCCGAATC
Prom_rbc-A1	TCGGGGGCCCCCGGGGGATTTGTTGCTAAAAGATAAAAATAAGTCG
Prom_rbc-A2	TCGGGCCCtcctCCGGGGATGCGGTTTTCCTCCAGCAAAAATGC
PepS_A0313-A2	ATCCCCGGaggaGGGCCCGAATGCCCAATCGCCGCCATTTTTTG
PepS_A0313-A3	AGTTCTTCTCCTTTGCTCATTCGTTGACACCCTCCCAAACACCC
PepS_A0500-A2	ATCCCCGGaggaGGGCCCGAATGTTTAATGGTCTCAAAGGGCTAC
PepS_A0500-A3	AGTTCTTCTCCTTTGCTCATACCAGGTACCTGTTGACAAGCGGC
Vector-A1	ATCCCCCCGGGGGCCCCCGAgccagaaccgttatgatgtcggc
Vector-A3	atgagcaaaggagaagaacttttc
Prom_trc_R-A2	TCGGGCCCtcctCCGGGGATggtgaattccatggtctgtttc
pPC-F	CGCACTCCCGTTCTGGATAATGTT

pPC-R	GGTCACGCTTTTCGTTGGGATCTT

Table S7. Oligonucleotides used in the generation of mutant strains of *Synechococcus* sp. PCC 7002.
